# *IRX1* suppresses breast cancer progression by inhibiting fatty acid de novo synthesis through downregulating *ACACA* expression

**DOI:** 10.1038/s41419-026-08913-9

**Published:** 2026-06-01

**Authors:** Wen-Bo Liu, Lin-Yue Hai, Yuan-Yuan Zhang, Hao-Ran Yue, Xiao-Feng Liu, Bo-Wen Liu, Xin Wang, Xu-Chen Cao, Yue Yu

**Affiliations:** 1 The First Department of Breast Cancer, Tianjin Medical University Cancer Institute and Hospital, National Clinical Research Center for Cancer, Tianjin, China; 2 Key Laboratory of Cancer Prevention and Therapy, Tianjin, China; 3 Tianjin’s Clinical Research Center for Cancer, Tianjin, China; 4 Key Laboratory of Breast Cancer Prevention and Therapy, Tianjin Medical University, Ministry of Education, Tianjin, China

**Keywords:** Breast cancer, Cancer metabolism

## Abstract

Breast cancer remains a major threat to women’s health and survival worldwide. Iroquois homeobox 1 (*IRX1*), a developmentally regulated transcription factor, acts as a tumor suppressor in several cancers. However, its mechanistic role in breast cancer remains elusive. In this study, we demonstrate that *IRX1* is downregulated in breast cancer tissues and cell lines due to promoter hypermethylation. *IRX1* functioned as a tumor suppressor both in vitro and in vivo. Mechanistically, IRX1 interacts with NME1 and promotes its nuclear localization. Subsequently, *NME1* facilitates the transcriptional regulation of *ACACA* by *IRX1*. The *IRX1*-*NME1* axis modulates de novo fatty acid synthesis and breast cancer progression by targeting *ACACA*. In conclusion, our findings reveal that the *IRX1- NME1*/*ACACA* axis plays a critical role in de novo fatty acid synthesis and breast cancer progression, providing new insights into gene regulatory interactions and highlighting its potential as a novel therapeutic target for breast cancer.

## Introduction

Breast cancer remains the most prevalent malignancy and the leading cause of cancer-related mortality among women worldwide [1]. It is a highly heterogeneous disease [2]. Although the therapeutic arsenal—including surgery, radiotherapy, chemotherapy, endocrine, targeted, and immunotherapy—has significantly improved local control and long-term survival [3], a substantial number of patients still have a poor prognosis. Therefore, a critical need remains to explore novel biomarkers to elucidate the molecular mechanisms driving breast cancer progression, enabling precise therapies and improved patient survival.

Many major genetic alterations driving human carcinogenesis involve transcription factors. Developmental transcription factors are key regulatory proteins governing embryonic development. Among them, the Iroquois homologous frame (*IRX*) gene family encodes a class of transcription factors containing a unique homeodomain. These factors are highly conserved from invertebrates to vertebrates, play crucial roles in development, and have been implicated in regulating several cancers [4–7]. As a member of this family, *IRX1* is involved in developmental processes and cellular differentiation and has emerged as a key factor in tumorigenesis. For instance, Jung et al. demonstrated that *IRX1* acts as a tumor suppressor by inhibiting tumor cell growth *via* cell cycle regulation [8]. Yu et al. identified that *IRX1* acts as a tumor suppressor gene in gastric cancer, whose epigenetic silencing enhances the tumorigenicity, and *IRX1* overexpression inhibits peritoneal spreading and distant metastasis by suppressing *BDKRB2*-dependent angiogenesis [9–11]. Furthermore, *IRX1* can influence tumor progression by modulating the TGF-β signaling pathway and NF-κB signaling pathway [12–14]. However, the role and potential mechanisms of *IRX1* in breast cancer remain inadequately explored.

Metabolic reprogramming is a hallmark of cancer. Fatty acid metabolism is pivotal for both energy supply and biosynthetic processes in tumor cells. It promotes tumorigenesis, progression, and metastasis by fulfilling the bioenergetic demands of rapid proliferation, supplying substrates for membrane biogenesis, and modulating signal transduction pathways [15, 16]. Acetyl-CoA Carboxylase Alpha (*ACACA*) is the rate-limiting enzyme in fatty acid synthesis. It catalyzes the carboxylation of acetyl-CoA to malonyl-CoA, the essential substrate for producing long-chain fatty acids such as palmitic acid. These lipids serve as precursors for diverse biomass, thereby promoting tumor malignancy [17, 18]. *ACACA* is highly expressed in cancers, including breast, colorectal, and prostate cancers, where it drives tumorigenic lipid synthesis and progression [19–21]. For example, Zhang et al. reported that reduced *ACACA* expression inhibits the malignant progression of prostate cancer by inhibiting mitochondrial potential [22]. Recent studies have revealed that *ACACA* expression is regulated at multiple levels, including transcriptional control by specific factors and post-transcriptional mechanisms such as m6A and m5C RNA modification [21, 23, 24]. However, the specific regulatory mechanisms controlling *ACACA* expression in breast cancer remain to be fully elucidated.

In this study, we demonstrate that IRX1, by transcriptionally repressing *ACACA*, inhibits fatty acid metabolism and malignant progression in breast cancer cells. Furthermore, we identified *NME1* as a key cofactor facilitating *IRX1*-mediated transcriptional repression of *ACACA*. Mechanistically, *IRX1* interacts with *NME1* and promotes its nuclear translocation. In turn, *NME1* enhances the transcriptional repression of *ACACA* by *IRX1*. Collectively, our results define a novel *IRX1-NME1*/*ACACA* regulatory axis that plays a critical role in breast cancer development.

## Materials and methods

### Clinical samples

Breast cancer tissue specimens were obtained from the Tianjin Medical University Cancer Institute and Hospital (TMUCIH). This study included a cohort of 20 breast cancer tissues and their matched adjacent non-tumor tissues. All tumor specimens were collected from newly diagnosed breast cancer patients prior to any therapeutic intervention. This study was approved by the Institutional Review Board of TMUCIH. Written informed consent was obtained from all participants.

### Immunohistochemistry (IHC)

Following deparaffinization and dehydration, antigen retrieval was performed by heating the tissue sections at 98 °C for 18 min in 0.01 M sodium citrate buffer, followed by natural cooling to room temperature. Endogenous peroxidase activity was quenched by incubation with 3% hydrogen peroxide for 10 min. Sections were then blocked with 5% bovine serum albumin (BSA) for 1 h at room temperature, followed by incubation with primary antibody overnight at 4 °C. After washing with phosphate-buffered saline (PBS), sections were incubated with horseradish peroxidase-conjugated secondary antibody for 1 h at room temperature. Signal development was performed using 3,3’-diaminobenzidine (DAB) substrate. Finally, sections were counterstained with hematoxylin, differentiated in acid alcohol, dehydrated, and mounted with neutral gum. The protein expression levels of IRX1, ACACA, and Ki-67 were evaluated using a semiquantitative score system based on the product of staining intensity and the percentage of positive cells. Staining intensity was scored as follows: 0 (negative), 1 (weak), 2 (moderate), and 3 (strong). The percentage of positive cells was scored as: 0 (0%), 1 (1–25%), 2 (26–50%), 3 (51–75%), and 4 (>75%). All sections were examined and scored independently by two experienced pathologists using light microscopy.

### Cell culture, plasmids and transfection

The normal human breast epithelial cell line MCF-10A and the breast cancer cell lines MDA-MB-231, MCF-7, T47D, and CAL51 were obtained from ATCC (American Type Culture Collection, Manassas, USA). MCF-10A cells were cultured in MCF-10A cell-specific medium (Procell, China). MCF-7 and MDA-MB-231 cells were cultured in DMEM (Gibco, USA), whereas T47D and CAL51 cells were cultured in RPMI-1640 (Gibco, USA). All media were supplemented with 10% fetal bovine serum (FBS, NEWZERUM, Australia) and 1% penicillin/streptomycin (Gibco, USA). All cell lines were cultured at 37 °C in a humidified incubator with 5% CO_2_.

*IRX1-, NME1-*, and *ACACA*-overexpression plasmids, *ACACA* siRNAs, and their corresponding controls were obtained from Keyybio (Shandong, China). A shRNA sequence targeting *IRX1* was designed by Tsingke Biotech (Beijing, China) and cloned into the pLKO vector to generate the *IRX1*-shRNA plasmid. Site-directed mutagenesis primers for the *ACACA* promoter region were synthesized by Tsingke Biotech (Beijing, China). The pGL3-*ACACA*-mut reporter plasmid was subsequently constructed using these primers. Transient transfections were performed using Lipofectamine 3000 reagent (Invitrogen, USA) according to the manufacturer’s instructions. To generate stable cell lines, MCF-7, CAL51, MDA-MB-231, and T47D cells were infected with lentiviruses. The lentiviruses for *IRX1* overexpression or knockdown were packaged in 293T cells using the GeneCopoeia Lentiviral Packaging Kit. The sequences of all siRNA oligonucleotides used are listed in Supplementary Table S1. The target sequences of the *IRX1*-specific shRNAs are provided in Supplementary Table S2. The primer sequences used for constructing the mutant *ACACA* luciferase reporter plasmid are detailed in Supplementary Table S3.

### Antibodies and reagents

The following primary antibodies were used: anti-IRX1 (cat. #YT2412, Immunoway, China), anti-NME1(cat. #11086-2-AP, proteintech, China), anti-ACACA (cat. #21923-1-AP, proteintech, China), anti-Flag (cat. #YM3001, Immunoway, China), anti-GST (cat. #2622S, Cell Signaling Technology, USA), anti-IgG (cat. # RS0002, Immunoway, China), anti-FASN (cat. #YT1683, Immunoway, China), anti-ACACB (cat. #YT7481, Immunoway, China), anti-SREBP1 (cat. #YT6055, Immunoway, China), anti-SREBP2 (cat. #YN0037, Immunoway, China), anti-ACSL3 (cat. #YN0828, Immunoway, China), anti-ACSL4 (cat. #YT8070, Immunoway, China), anti-GAPDH (cat. #10494-1-AP, proteintech, China), anti-β-actin (cat. #20536-1-AP, proteintech, China), anti-HDAC1 (cat. #2062S, Cell Signaling Technology, USA). The DNA methyltransferase inhibitor decitabine (5-Aza-2′-deoxycytidine) was purchased from MedChemExpress (Monmouth Junction, NJ, USA).

### DNA isolation and bisulfite sequencing PCR (BSP)

Genomic DNA was isolated from human cell lines and tissue specimens using a genomic DNA isolation kit (Thermo Scientific, USA). Bisulfite conversion of DNA was performed using the EpiTect bisulfite kit (Qiagen, USA). The bisulfite-converted was used as a template to amplify a 334 bp fragment encompassing the *IRX1* promoter region (−770 to −437). The PCR products were purified using the EasyPure Quick Gel Extraction Kit (Transgene, China), cloned into a pGEM-T easy vector (Promega, USA), and transformed into competent cells. For each sample, eight randomly selected bacteria were picked and subjected to Sanger sequencing. Methylation status at each CpG site was determined by analyzing the cytosine (C, methylated) to thymine (T, unmethylated) conversion ratio in the sequencing chromatograms. The primers used for BSP are listed in Supplementary Table S4.

### Proliferation assays

For the CCK8 assay, cells were seeded in 96-well plates at a density of 2 × 10^3^ cells per well. On days 0, 1, 2, 3, and 4, the culture medium was replaced with DMEM containing 10% CCK-8 reagent and incubated for 1 h. The absorbance at 450 nm was then measured using a microplate reader.

For colony formation assay, 1 × 10^3^ cells were seeded in 6-well plates and cultured for 10–14 days until distinct colonies were visible. The colonies were then fixed with 4% formaldehyde for 15 min and stained with 1% crystal violet for 30 min. After washing and air-drying, images were captured, and colonies containing more than 50 cells were manually counted.

The EdU assay was performed using the EdU detection kit (RiboBio, China) according to the manufacturer’s protocol. The percentage of EdU-positive cells was quantified by counting cells in five random fields of view per well using a fluorescence microscope (Leica, Germany).

### Cell migration assay

Cell migration was assessed using a Transwell chamber (BD Biosciences, USA). Briefly, 6 × 10^4^ cells in serum-free medium were seeded into the upper chamber, while the lower chamber was filled with 500 μl of medium containing 10% FBS as a chemoattractant. After incubation (6–8 h for MAD-MB-231; 12–36 h for MCF7, T47D, and CAL51 cells), the cells on the upper surface of the membrane were removed with a cotton swab. Cells that had migrated to the lower surface were fixed with 4% formaldehyde and stained with 1% crystal violet for 30 min. The stained cells were imaged under a light microscope at 10× magnification. The number of migrated cells was quantified by counting five random fields per membrane.

For the wound healing assay, cells were seeded in 6-well plates and cultured until they reached 80−90% confluency. A uniform wound was created in each well by scratching the cell monolayer with a sterile 10-µl pipette tip. The cells were then gently washed with PBS to remove debris and cultured in serum-free medium to minimize the influence of cell proliferation on migration. Images of the wounds were captured at 0 h and 24 h after scratching using a light microscope equipped with a camera. The wound area at each time point was measured using ImageJ software. The relative migration rate was calculated as the percentage of wound closure compared to the 0 h time point.

### RNA isolation and quantitative reverse transcription PCR (RT-qPCR)

Total RNA was isolated using the Rapid cellular RNA extraction kit (Sparkjade, China) and reverse-transcribed into cDNA with the TransScript cDNA Synthesis system (TransGen, China). Quantitative RT-PCR was performed with One-Step RT-qPCR SuperMix (TransGen, China) following the manufacturer’s protocols. Gene expression levels were normalized to *GAPDH* and quantified via the 2−ΔΔCt method. All specific sequences are listed in Supplementary Table S5.

### Western blot

Cells were harvested and lysed in RIPA lysis buffer (Solarbio, Beijing, China) supplemented with 1 mM PMSF protease inhibitor. The protein concentration was quantified using the BCA Protein Assay kit (Thermo Fisher Scientific) according to the manufacturer’s instructions. Equal amounts of protein (20−40 µg) were separated by SDS-PAGE and transferred onto polyvinylidene fluoride membrane (Millipore, Bedford, MA, USA). The membranes were then blocked with 5% non-fat milk for 1 h at room temperature, followed by incubation with the primary antibodies overnight at 4 °C. After washing with TBST three times (10 min each), membranes were incubated with horseradish peroxidase (HRP)-conjugated secondary antibodies for 1 h at room temperature. Protein bands were visualized using an enhanced chemiluminescence (ECL) detection reagent (Millipore, USA) and imaged with a chemiluminescence imaging system. β-actin or GAPDH was used as a loading control for normalization of protein levels. The band intensity was quantified using ImageJ software.

### Immunofluorescence (IF)

Cells were seeded onto sterile glass coverslips placed in 24-well plates at a density of 5 × 10^4^ per well and cultured overnight. The following day, cells were washed three times with PBS and fixed with 4% paraformaldehyde in PBS for 30 min at room temperature. Cells were then permeabilized and blocked with PBS containing 0.1% Triton X-100 and 3% BSA for 1 h at room temperature. Subsequently, cells were incubated with primary antibodies diluted in blocking buffer (1:500−1:1000) at 4 °C overnight. After washing, cells were incubated with appropriate species-specific fluorescently conjugated secondary antibody (1:500 dilution) for 1 h at room temperature, protected from light. Nuclei were counterstained with Hoechst 33342 (1 µg/ml) for 10 min at room temperature. Finally, coverslips were mounted onto glass slides using antifade mounting medium and visualized under a Zeiss fluorescence microscope.

Multiplex immunofluorescence staining of paraffin-embedded tissue sections was performed using the TSAPLus Fluorescence Triple Labeled Quadruple Staining Kit (Servicebio, Wuhan, China; Cat# G1236) according to the manufacturer’s protocol. The procedure included antigen retrieval using citrate buffer (pH 6.0) at 95 °C for 15 min, blocking with 3% BSA for 30 min at room temperature, and sequential incubation with primary antibodies (diluted 1:200) at 4 °C overnight and corresponding fluorophore-conjugated secondary antibodies for 1 h at room temperature. Cell nuclei were counterstained with 4’, 6-diamidino-2-phenylindole (DAPI) contained in the antifade mounting medium. High-resolution multichannel fluorescence images were acquired by Servicebio technicians using a Nikon Eclipse C1 confocal microscope (Nikon, Japan) equipped with appropriate filter sets. Image analysis was performed using Image-Pro Plus software (Media Cybernetics, USA).

### Co-immunoprecipitation (Co-IP) and protein identification

Breast cancer cells were washed twice with ice-cold PBS and lysed in IP lysis buffer (Thermo, Massachusetts, USA) supplemented with protease inhibitor cocktail for 30 min on ice. Cell lysates were pre-cleared with protein A/G beads for 1 h at 4°C, then incubated overnight at 4 °C with specific antibodies against IRX1, NME1, Flag, HA, or normal IgG (negative control). The following day, 40 µl of pre-washed protein A/G magnetic beads (Vazyme, China) were added and incubated for 2 h at 4°C with gentle rotation. Beads were washed three times with ice-cold lysis buffer, followed by two washes with PBS to minimize non-specific binding. For Co-IP validation, immunoprecipitated proteins were eluted in 2×SDS loading buffer by boiling at 95 °C for 10 min and analyzed by western blot as described previously. For mass spectrometry analysis, immunoprecipitated proteins were separated by SDS-PAGE and stained with Coomassie Brilliant Blue solution (Solarbio, China). Proteins identification was performed by liquid chromatography-tandem mass spectrometry on a Q Exactive HF-X instrument by Novogene Co., Ltd (China). For IP-MS experiments, MCF7 cells overexpressing IRX1-FLAG were subjected to immunoprecipitation using FLAG M2 magnetic beads with normal IgG as a negative control. Proteins identified in the IgG control group were excluded from the experimental group results to eliminate false-positive interactions.

### GST-pull down

To obtain fusion proteins, BL21(DE3) cells were transformed with plasmids carrying GST-IRX1 and His-NME1. GST and GST-IRX1 fusion proteins (100 µg each) were then captured on 50 µL of glutathione agarose beads under gentle agitation at 4 °C for 1 h. Following three washes with PBST, 100 µg of the His-NME1 fusion protein was added to the beads prebound with GST or GST-IRX1, and the mixture was incubated at 4 °C for 4 h with gentle mixing. After three additional washes with PBST, the bound proteins were eluted using elution buffer (10 mM reduced glutathione in PBS, pH 8.0) and subsequently analyzed by western blotting.

### Electrophoretic mobility shift assay (EMSA)

DNA probes encompassing the putative IRX1 binding site in the *ACACA* promoter region were designed. 5’-biotin-labeled wild-type and mutant probes were synthesized by Tsingke Biotech (China). The probe sequences are provided in Supplementary Table S6. Nuclear extracts were prepared for breast cancer cells using the Nuclear and Cytoplasmic Protein Extraction Kit (Beyotime, China) according to the manufacturer’s instructions. For supershift electrophoretic mobility shift assay (EMSA) assays, nuclear extracts were pre-incubated with anti-IRX1 antibody or normal IgG (negative control) for 30 min at 4 °C prior to adding the biotin-labeled probe, using the chemiluminescence EMSA kit (Beyotime, China). Protein-DNA binding reactions were separated on 6% non-denaturing polyacrylamide gels in 0.5× TBE buffer at 100 V for 60−90 min at 4 °C. Following electrophoresis, the samples were transferred to a positively charged nylon membrane (Beyotime, China) using a semi-dry transfer system. UV crosslinking was used to bind the DNA oligomers to the membrane, and the chemiluminescent imaging system was employed to detect the labeled probes.

### Oil Red O staining

Transfected cells were fixed with 4% paraformaldehyde (PFA) in PBS for 30 min at room temperature. After washing twice with PBS, cells were incubated with freshly prepared Oil Red O working solution (Solarbio, China) for 15 min at room temperature. Cells were then counterstained with Mayer’s hematoxylin solution for 1−2 min, washed with running tap water for bluing, and mounted with aqueous mounting medium. Images were captured using an inverted light microscope (Nikon, Japan) at 200× magnification.

### Measurement of triglyceride (TG) and cholesterol

Cellular total cholesterol and triglyceride levels were quantified using a Cholesterol Assay Kit (Beyotime, China) and Triglyceride Assay Kit (Beyotime, China) according to the manufacturer’s instructions. Briefly, 1.5 × 10^6^ cells were harvested and lysed with the appropriate lysis buffer provided in the kits. The lysates were centrifuged at 12,000 × *g* for 10 min at 4 °C to remove cellular debris. 50 µl of cell supernatant was mixed with 150 µl of working solution in 96-well plates and incubated at 37 °C for 30 min protected from light. The absorbance was measured at 570 nm using a microplate reader (BioTek, USA), concentrations were calculated based on standard curves. All samples were analyzed in triplicate.

### Measurement of malonyl coenzyme A (Mal-CoA), palmitate, and ceramide content

Cellular concentrations of malonyl-CoA, free palmitate, and ceramide were quantified using commercial enzyme-linked immunosorbent assay (ELISA) kits. Briefly, 2 × 10^6^ cells were harvested and resuspended in 100 µl of PBS. Cell samples were sonicated on ice using a probe sonicator (Scientz, China) at 200 W output (3-s pulses followed by 10-s intervals, repeated 30 times) to ensure complete cell lysis while preventing sample heating. The lysates were then centrifuged at 12,000 × *g* for 15 min at 4 °C to remove insoluble debris. Malonyl-CoA, palmitate, and ceramide levels were measured in the supernatant using the specific Human Malonyl-CoA ELISA Kit, Palmitic acid ELISA Kit and ceramide ELISA Kit (Enzyme Immunoassay Technology Co., Ltd, China) according to the manufacturer’s protocols. The absorbance was measured at 450 nm using a microplate reader (BioTek, USA). Concentrations were calculated based on standard curves. All samples were analyzed in triplicate.

### Dual-luciferase reporter assay

Cells were seeded in a 12-well plate at a density of 5 × 10^4^ cells per well and cultured for 24 h to reach 70−80% confluency. Cells were co-transfected with 600 ng of pGL3-*ACACA* promoter firefly luciferase reporter plasmid, 600 ng of *IRX1* expression plasmid (or empty vector control), and 60 ng of pRL-TK Renilla luciferase control plasmid using Lipofectamine 3000 reagent (Invitrogen). The transfection mixture was replaced with fresh complete medium after 6 h, and cells were further cultured for 48 h. Luciferase activities were measured using the Dual Luciferase Reporter Gene Assay Kit (Transgene) according to the manufacturer’s protocol. Firefly luciferase activity was normalized to Renilla luciferase activity for each sample.

### Xenograft

Thirty-five 5-week-old female NSG mice were randomly assigned to different experimental groups for subcutaneous xenograft models. A suspension containing 8 × 10^6^ cells mixed with 5% Matrigel (BD Biosciences, USA) was injected subcutaneously into the right flank of female NSG mice. Mice were monitored for 6 weeks, and tumor growth was measured every 5 days using digital calipers. At the experimental endpoint, final tumor volumes were recorded. Tumors were excised, photographed, and fixed in 4% paraformaldehyde for 24 h at 4 °C for subsequent histological analysis. Tumor volume was calculated using the formula: Volume (mm^3^) = length × width^2^× 1/2. Paraffin-embedded tumor sections were stained with hematoxylin and eosin (H&E) for histological evaluation and processed for immunohistochemical analysis using standard protocols. Each experimental group consisted of five mice (*n* = 5). All animal procedures were approved by the Ethics Committee of the Tianjin Medical University Cancer Hospital and performed in accordance with NIH Guide for the Care and Use of Laboratory Animals.

### Statistical analysis

All statistical analyzes were performed using GraphPad Prism software (GraphPad Software, CA, USA). Comparisons between the two groups were analyzed using unpaired two-tailed Student’s *t*-test. For comparisons among three or more groups, one-way Analysis of Variance (ANOVA) followed by Tukey’s post hoc test was employed. Correlation analyzes were performed using the Pearson correlation coefficient. Data are presented as mean ± standard error of the mean from at least three independent experiments. Statistical significance was defined as *p* < 0.05.

## Results

### Downregulation of IRX1 expression is associated with poor prognosis in breast cancer

Analysis of IRX family member expression using the Oncomine database (https://www.oncomine.org/) revealed significantly lower mRNA levels of *IRX1*, *IRX2, IRX4*, and *IRX6* in breast cancer tissues compared with normal tissues (Figure S1A). Consistent results were obtained from the GEPIA database (http://gepia.cancer-pku.cn/), confirming the downregulation of these IRX genes in breast cancer (Fig. S1B). Prognostic analysis using the Kaplan–Meier Plotter database (http://kmplot.com/analysis) demonstrated that low expression levels of IRX1, and IRX2, and IRX4 were significantly associated with poorer overall survival in breast cancer patients (Fig. S1C). Given these findings and the limited existing literature on IRX1 in breast cancer, we selected IRX1 for further investigation.

**Fig. 1:**
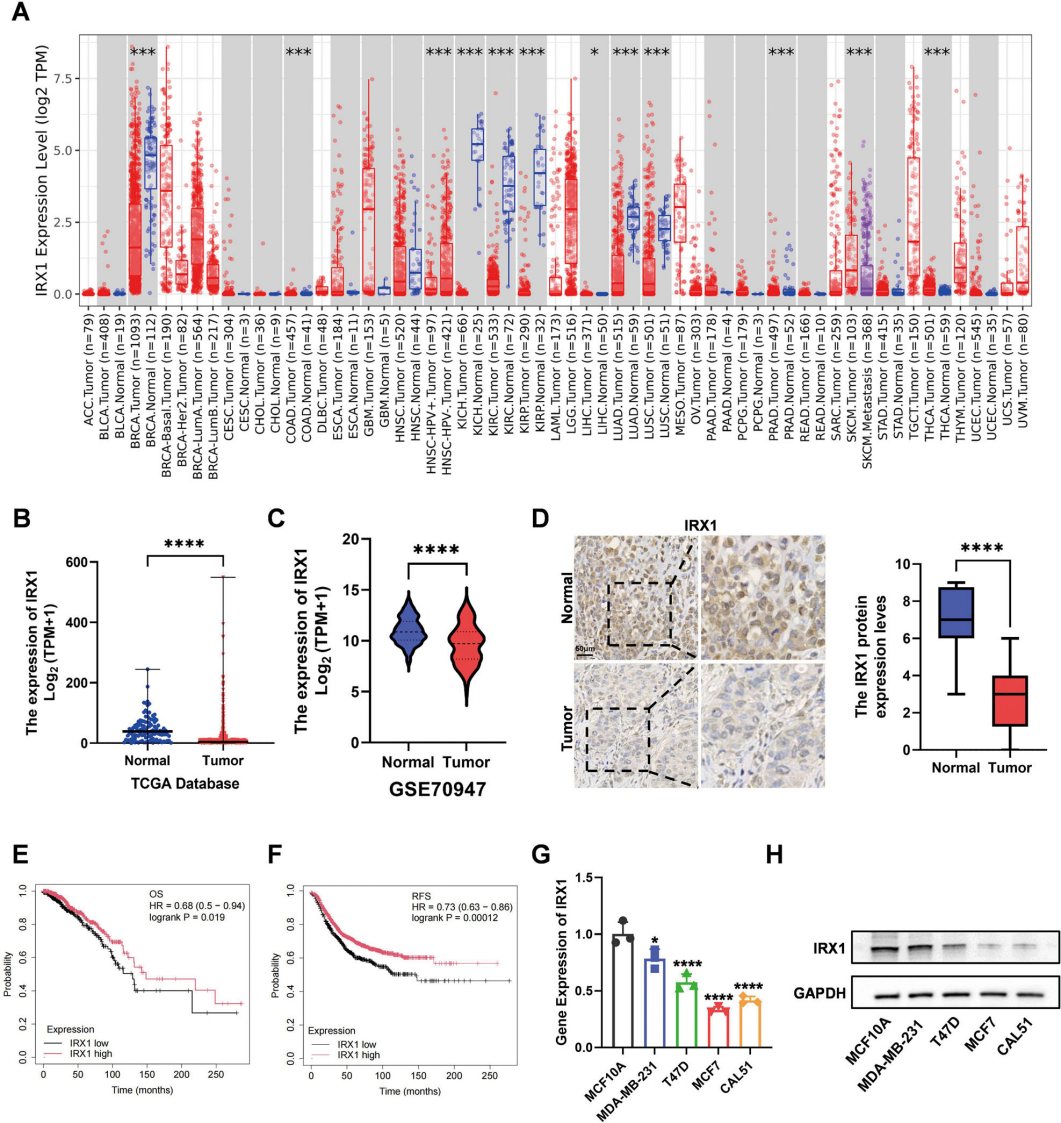
Downregulation of *IRX1* expression is associated with poor prognosis in breast cancer. **A** The *IRX1* expression level in various human cancers analyzed by Timer database. The *IRX1* expression level in breast cancer tissues and normal tissues from TCGA (**B**) and GSE70947 (**C**) database. **D** The protein expression level of IRX1 in breast cancer tissues and paired normal tissues determined by immunochemical analysis. Scale bar, 50 μm. Kaplan–Meier plotter analysis of overall survival (**E**) and recurrence-free survival (**F**) in breast cancer patients with different *IRX1* expression levels from the TCGA database. The *IRX1* expression level in breast cancer cell lines detected by RT-qPCR (**G**) and western blot (**H**). *P < 0.05, **P < 0.01, ***P < 0.001 or ****P < 0.0001.

Pan-cancer analysis via the TIMER database (http://timer.cistrome.org/) indicated that *IRX1* expression was significantly downregulated in a majority of cancer types, including breast cancer, compared to corresponding normal tissues (Fig. 1A). Furthermore, analysis of transcriptomic data from The Cancer Genome Atlas (TCGA) and Gene Expression Omnibus (GEO) databases confirmed that *IRX1* mRNA expression was significantly reduced in breast cancer tissues compared to normal breast tissues (Fig. 1B, C). Immunohistochemical analysis of 20 paired clinical specimens (tumor and adjacent normal tissue) revealed significantly lower IRX1 protein levels in breast cancer tissues, corroborating the bioinformatic findings (Fig. 1D). Kaplan–Meier survival analysis further showed that high *IRX1* expression was significantly associated with favorable overall survival (OS) and recurrence-free survival (RFS) in breast cancer patients (Fig. 1E, F). Finally, we assessed IRX1 expression in a panel of breast cell lines by RT-qPCR and western blot. Compared to the normal breast epithelial cell line MCF10A, IRX1 was downregulated in all breast cancer cell lines (MDA-MB-231, CAL-51, MCF-7, and T47D). Notably, IRX1 levels were relatively higher in MDA-MB-231 and T47D cells but substantially lower in MCF-7 and CAL-51 cells (Fig. 1G, H). Taken together, these results demonstrate that *IRX1* is consistently downregulated in breast cancer tissues and cell lines, and that low IRX1 expression is associated with poor prognosis in breast cancer patients.

### *IRX1* expression is regulated by promoter DNA methylation

**Fig. 2:**
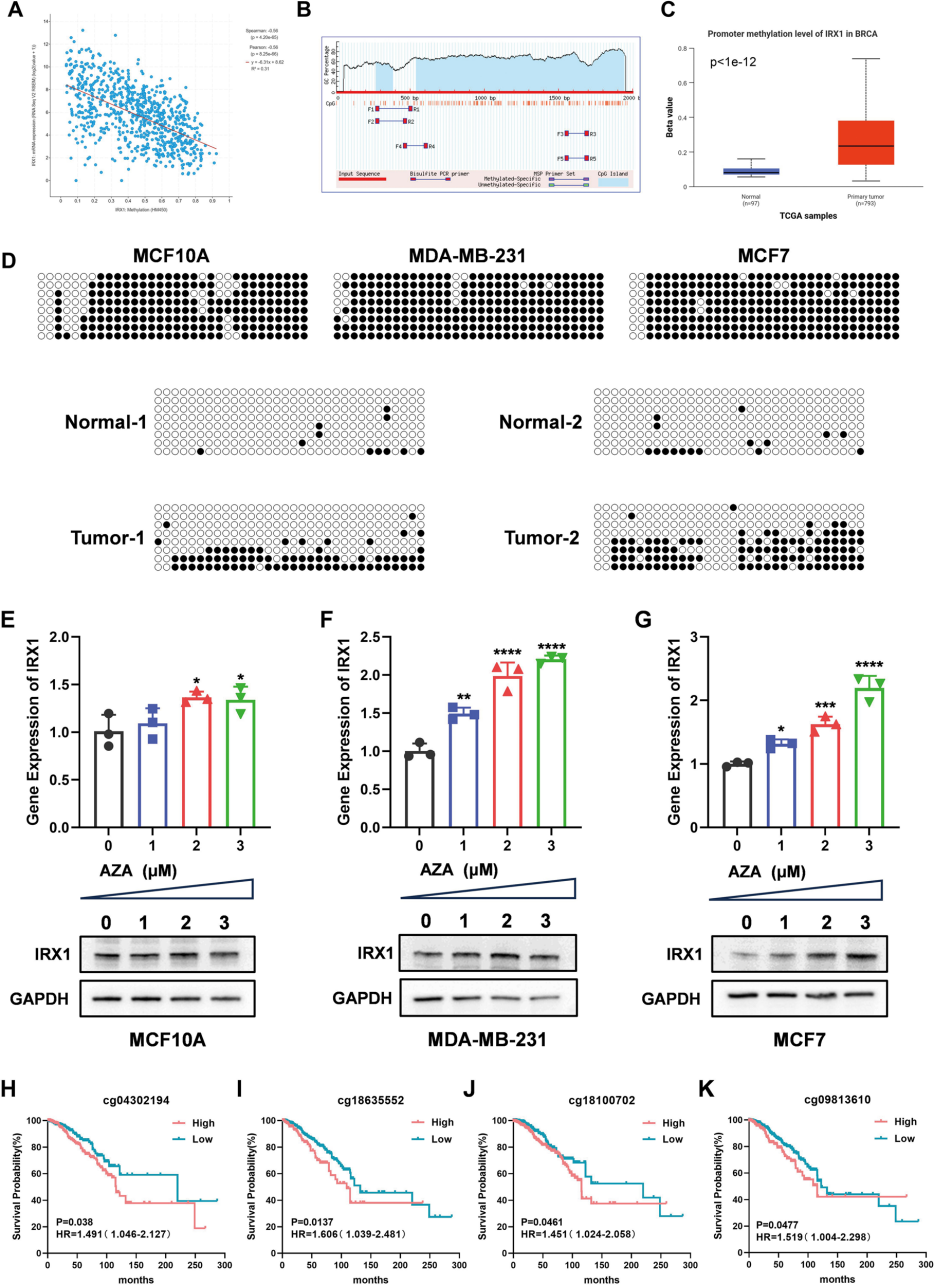
*IRX1* expression is regulated by its promoter DNA methylation. **A** The correlation between *IRX1* expression and its promoter DNA methylation analyzed by cBioPortal. **B** A CpG island on the *IRX1* promoter region predicted by the Methprimer. **C** The level of *IRX1* promoter DNA methylation was analyzed in normal tissue and breast cancer using UALCAN. **D** The DNA methylation status of *IRX1* promoter determined by BSP sequencing in breast cancer cell lines and tissues. The *IRX1* expression level in MCF 10 A (**E**), MDA-MB-231 (**F**), and MCF-7 (**G**) cell lines detected by RT-qPCR (upper) and western blot (lower) after treated with AZA. **H**–**K** Kaplan–Meier analyzed the correlation between the methylation level of CpG locus in the *IRX1* promoter region and the overall survival of breast cancer patients. *P < 0.05, **P < 0.01, ***P < 0.001 or ****P < 0.0001.

Previous studies have established a strong association between *IRX1* expression and DNA methylation [25, 26]. Using cBioPortal (http://www.cbioportal.org/), we analyzed the correlation between *IRX1* expression and its promoter methylation status and observed a significant negative correlation (Fig. 2A). A CpG island was predicted within the *IRX1* promoter region using MethPrimer (http://www.urogene.org/methprimer, Fig. 2B). Analysis of *IRX1* promoter methylation levels in normal tissues and breast cancers using UALCAN (https://ualcan.path.uab.edu/) showed significantly higher methylation in breast cancer samples (Fig. 2C). Bisulfite sequencing PCR (BSP) was performed to assess the *IRX1* promoter methylation status. The results demonstrated higher methylation levels in breast cancer cell lines. Similarly, breast cancer tissues exhibited higher *IRX1* promoter methylation compared to paired adjacent normal tissues (Fig. 2D). To further validate this regulatory mechanism, breast cancer cells were treated with the DNA methyltransferase (DNMT) inhibitor 5-aza-2’-deoxycytidine (AZA) at concentrations of 1.0, 2.0, and 3.0 µM. Following 4 days of treatment, RT-qPCR and western blot revealed that *IRX1* expression was restored in a dose-dependent manner by AZA in the highly methylated cell lines MCF-7 and MDA-MB-231, but only marginally in the minimally methylated MCF-10A cells (Fig. 2E–G). These results indicate that *IRX1* expression is inversely correlated with the methylation status of its CpG islands. We extracted *IRX1* promoter methylation data from the TCGA-BRCA database and analyzed the correlation between *IRX1* promoter methylation and patient survival. The results revealed that hypermethylation of four CpG sites within the *IRX1* promoter region was associated with poorer OS (Fig. 2H–K).

### *IRX1* inhibits the malignant progression of breast cancer in vitro and in vivo

**Fig. 3:**
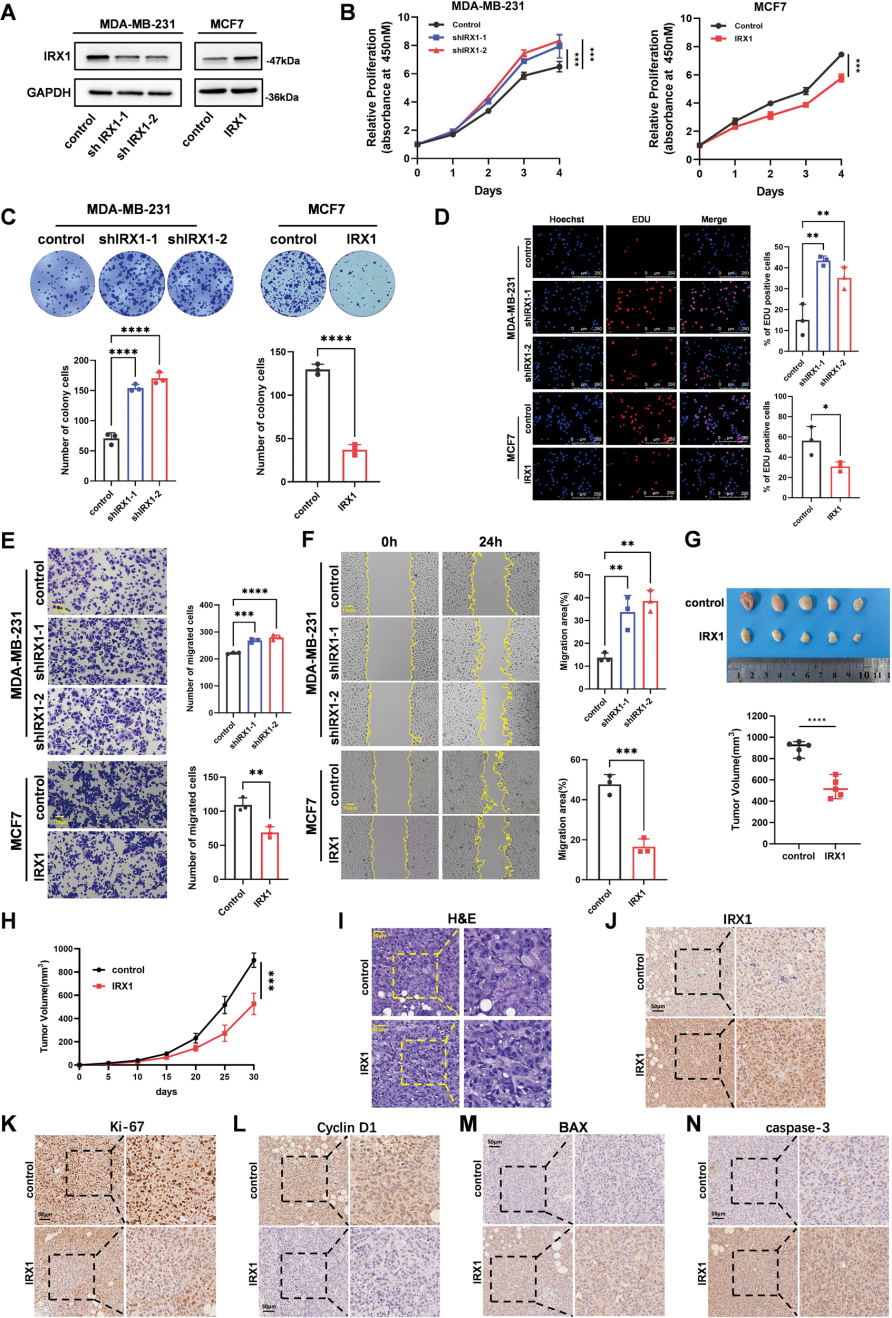
*IRX1* inhibits breast cancer proliferation and migration in vitro. **A** The expression of IRX1 in *IRX1*-knockdown MDA-MB-231, or *IRX1*-overexpressing MCF-7 cells, as well as control cells determined by western blot. CCK8 (**B**), colony formation (**C**), and EdU assays (**D**) were performed to assess the effect of *IRX1* on breast cancer proliferation. Scale bar, 250 μm (**D**). Transwell (**E**) and wound healing (**F**) assays were performed to assess the effect of *IRX1* on breast cancer migration. Scale bar, 100 μm (**E**, **F**). *P < 0.05, **P < 0.01, ***P < 0.001 or ****P < 0.0001. **G**, **H** Representative images, tumor volume and growth curves of subcutaneous xenografts in NSG mice derived from *IRX1*-overexpressing and control cells. (*n* = 5). HE staining (**I**) of mouse tumor tissue sections and the expression levels of IRX1 (**J**), Ki-67 (**K**), Cyclin D1 (**L**), BAX (**M**) and Caspase-3 (**N**) was assessed by immunohistochemical staining. Scale bar, 50 μm (**I**–**N**). *P < 0.05, **P < 0.01, ***P < 0.001 or ****P < 0.0001.

To investigate the functional role of *IRX1* in breast cancer progression, we established stable *IRX1-*overexpressing MCF7 and CAL51 cells, and *IRX1*-knockdown MDA-MB-231 cells and T47D cells, as well as the control cells. Successful modulation of IRX1 expression was confirmed by RT-qPCR and western blot (Figs. 3A and S2A–C). Functional assays were performed to assess the impact of *IRX1* on cell proliferation. CCK8, colony formation and EdU assays consistently demonstrated that *IRX1* overexpression significantly attenuated cell proliferation, reduced colony-forming capacity, and decreased the EdU-positive rate compared to control cells. Conversely, *IRX1* knockdown enhanced these proliferative capacities (Figs. 3B–D and S2D–F). These results indicate that *IRX1* acts as a suppressor of breast cancer cell proliferation. The effect of *IRX1* on cell migration was evaluated using wound healing and Transwell assays. *IRX1* overexpression markedly inhibited the migratory ability of MCF7 and CAL51 cells, while *IRX1* knockdown promoted the migration of MDA-MB-231 and T47D cells (Figs. 3E, F and S2G, H), demonstrating that *IRX1* suppresses the migration of breast cancer cells. We further validated the tumor-suppressive role of *IRX1* using a xenograft mouse model. NSG mice were subcutaneously injected with *IRX1*-overexpressing MCF7 cells and control cells (*n* = 5). Tumor volume was significantly reduced in the *IRX1*-overexpressing group compared to controls (Fig. 3G, H). In addition, mice injected with *IRX1*-knockdown MDA-MB-231 cells (*n* = 5) showed significantly increased tumor volume compared to the control group (Fig. S2I). Histological analysis of tumor sections via H&E staining revealed that *IRX1* overexpression suppressed tumor cell proliferation (Fig. 3I). IHC staining results of mouse tumor tissues showed that the expression levels of proliferation-related markers Ki-67 and Cyclin D1 were significantly downregulated in the *IRX1* overexpression group, while the expressions of apoptosis-related markers BAX and Caspase-3 proteins were upregulated (Fig. 3J–N).

### *IRX1* suppresses lipid metabolism in breast cancer cells

**Fig. 4:**
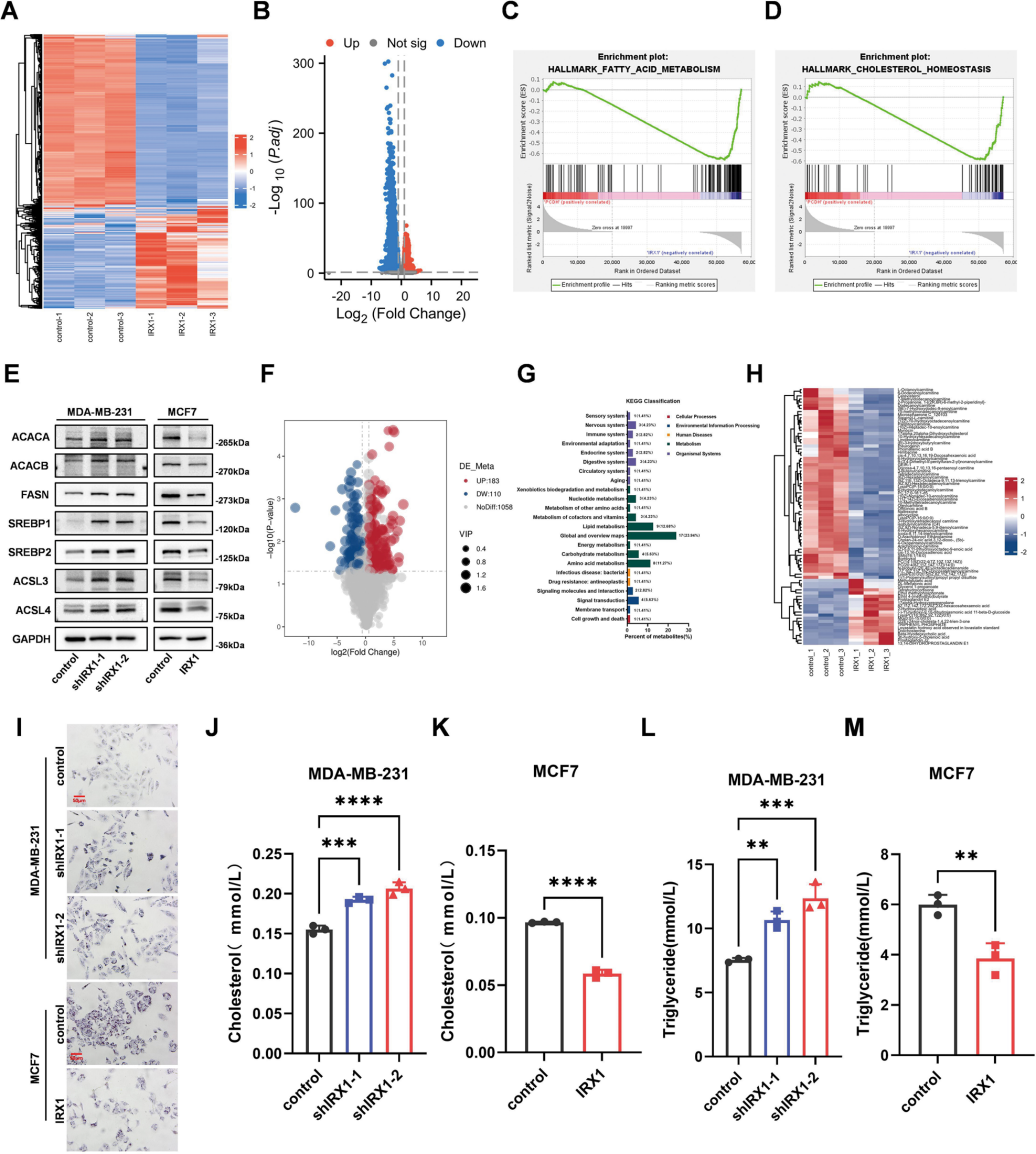
*IRX1* suppresses lipid metabolism in breast cancer cells. Heatmap (**A**) and volcano plot (**B**) analyses of differentially expressed genes between *IRX1-*overexpressing MCF7 and control cells. **C**, **D** GSEA of the differentially expressed genes. **E** The effect of IRX1 on the expression of lipid metabolism-related genes determined by western blot. **F** non-targeted metabolomics analysis was performed to determine the effect of *IRX1* on cellular metabolites. **G** KEGG classification of differential metabolites. **H** Heat map of differential products of lipid metabolism in *IRX1*-overexpressing and control cells. **I** The lipid droplets abundance of *IRX1*-overexpressing MCF7 or *IRX1*-knockdown MDA-MB-231 cells, as well as control cells determined by Oil red O staining. Scale bar, 50 μm. Cholesterol (**J**, **K**) and triglyceride (**L**, **M**) contents were determined in *IRX1*-overexpressing MCF7, *IRX1*-knockdown MDA-MB-231 and control cells. *P < 0.05, **P < 0.01, ***P < 0.001 or ****P < 0.0001.

To investigate the mechanism by which *IRX1* inhibits breast cancer progression, we performed RNA sequencing (RNA-seq) on *IRX1-*overexpressing MCF7 cells and control cells. Transcriptome analysis identified 2888 upregulated and 4302 downregulated genes in *IRX1-*overexpressing MCF7 cells compared to control cells (Fig. 4A, B). Gene Set Enrichment Analysis (GSEA) of the differentially expessed genes revealed significant enrichment in cholesterol homeostasis and fatty acid metabolism pathways (Fig. 4C, D). Gene Ontology (GO) and Kyoto Encyclopedia of Genes and Genomes (KEGG) analyzes similarly indicated enrichment in metabolic pathways (Fig. S3A, B). The expression levels of key lipid metabolism enzymes were examined by Western blot. The results showed that FASN, ACACA, ACACB, SREBP1, SREBP2, ACSL3, and ACSL4 were markedly decreased in *IRX1*-overexpressing cells, but increased in *IRX1*- knockdown cells (Figs. 4E and S3C).

To further validate the role of *IRX1* in lipid metabolism, we performed non-targeted metabolomics analysis, which revealed distinct metabolite profiles between *IRX1-*overexpressing and control cells (Fig. 4F). KEGG pathway analysis of differential metabolites showed their predominant clustering in the metabolic pathway (Fig. 4G). Specifically, fatty acid-derived metabolites and steroids were significantly reduced in *IRX1-*overexpressing cells compared to the control cells (Fig. 4H). Oil red O staining confirmed that lipid droplet abundance was markedly reduced in *IRX1*-overexpressing cells, but increased in *IRX1*-knockdown cells compared to their control cells (Figs. 4I and S3D). Consistent with these findings, cellular triglycerides and total cholesterol levels were significantly decreased upon *IRX1* overexpression (Figs. 4J–M and S3E, F). Collectively, these results demonstrate that IRX1 suppresses lipid metabolism in breast cancer cells.

### *IRX1* transcriptionally represses *ACACA* expression

**Fig. 5:**
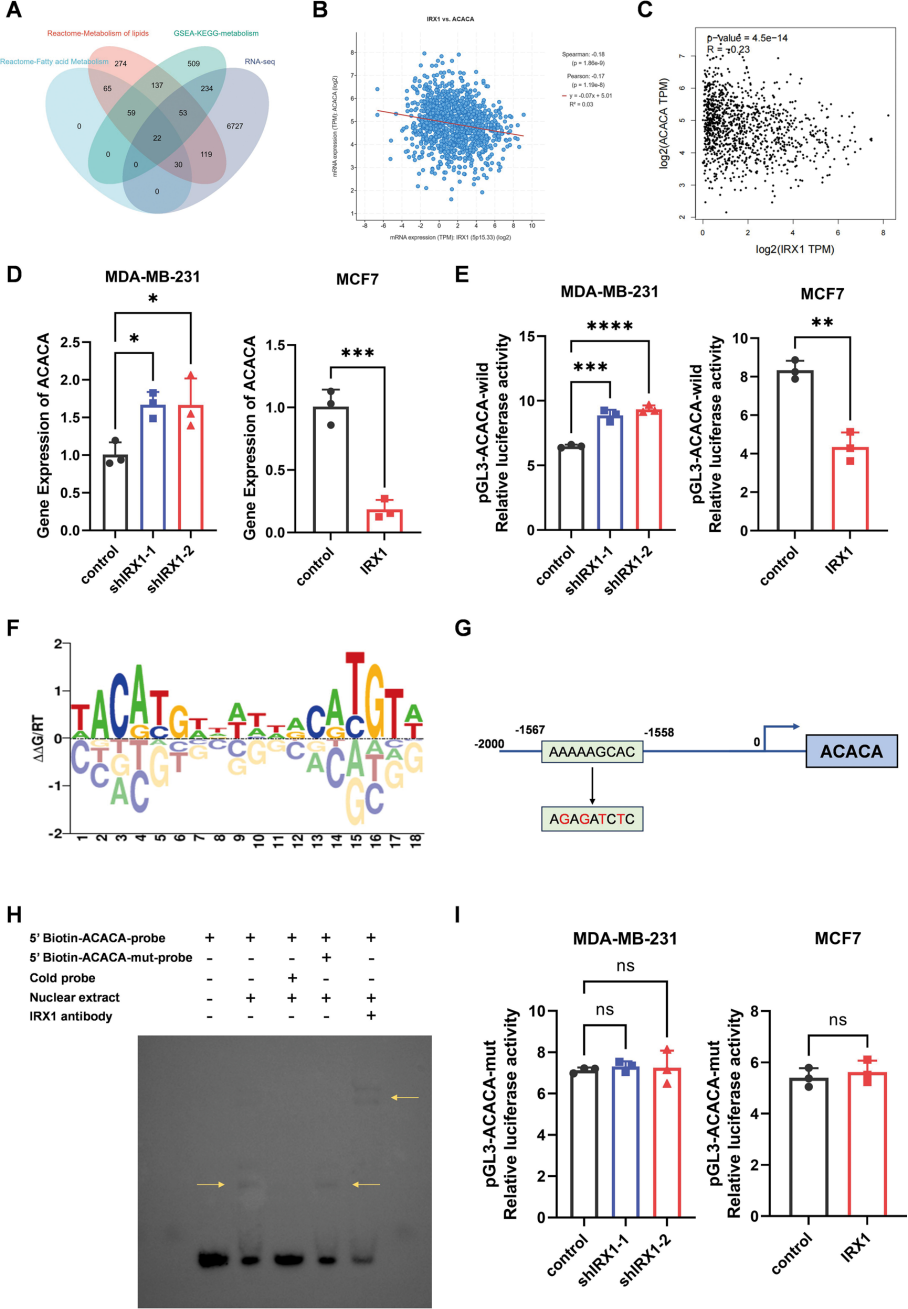
*IRX1* transcriptionally represses *ACACA* expression. **A** Integration of lipid metabolism-related gene sets and RNA-seq data identified putative downstream targets regulated by *IRX1*. The correlation between *IRX1* and *ACACA* was analyzed by cBioPortal (**B**) and GEPIA (**C**) database. **D** The mRNA expression level of *ACACA* in *IRX1-*knockdown MDA-MB-231 or *IRX1*-overexpressing MCF7 cells, as well as the control cells was determined by RT-qPCR. **E** The effect of *IRX1* on *ACACA* promoter activity was determined by dual-luciferase analysis. **F**, **G** A putative IRX1 binding site on *ACACA* promoter region analyzed by The Human Transcript Factors database. **H** EMSA of biotinylated wild-type or mutant *ACACA* probes in the presence of the IRX1 antibody. **I** The effect of *IRX1* on mutant *ACACA* promoter activity was determined by dual-luciferase analysis. *P < 0.05, **P < 0.01, ***P < 0.001 or ****P < 0.0001.

To elucidate the mechanism by which *IRX1* regulates lipid metabolism, we integrated lipid metabolism-related gene sets from the Reactome and GSEA database with our RNA-seq data. This analysis identified *ACACA* as a key candidate in the overlapping gene set, suggesting it as a potential target of *IRX1* (Fig. 5A). Analysis of the cBioPortal and GEPIA databases confirmed a significant negative correlation between *IRX1* and *ACACA* expression levels in breast cancer samples (Fig. 5B, C). RT-qPCR analysis indicated that the mRNA expression of *ACACA* was increased in *IRX1-*knockdown MDA-MB-231 cells and decreased in *IRX1-*overexpressing MCF7 cells compared to control cells (Fig. 5D). Next, we performed a dual-luciferase reporter assay to test whether *IRX1* transcriptionally regulates *ACACA*. The results indicate that *IRX1* overexpression significantly suppressed the activity of the *ACACA* promoter luciferase reporter (Fig. 5E). To further characterize *ACACA* as a direct transcriptional target of *IRX1*, we analyzed the *IRX1* binding motif using The Human Transcript Factors database. Scanning the *ACACA* promoter sequence revealed a putative *IRX1* binding site (5′-AAAAAGCAC-3′) located at positions −1567 to −1558 relative to the transcription start site (Fig. 5F, G). EMSA demonstrated that *IRX1* specifically binds to the 5′-AAAAAGCAC-3′ sequence in the *ACACA* promoter (Fig. 5H). Next, we constructed an *ACACA* promoter reporter with a mutated *IRX1* binding site (5′-AGAGATCTC-3′). In contrast to the wild-type promoter reporter, *IRX1* failed to suppress the activity of this mutant reporter (Fig. 5I). Collectively, these results demonstrate that *IRX*1 directly binds to the *ACACA* promoter and function as a transcriptional repressor to downregulate *ACACA* expression.

### IRX1 promotes the nuclear localization of NME1 to co-repress ACACA transcription

**Fig. 6:**
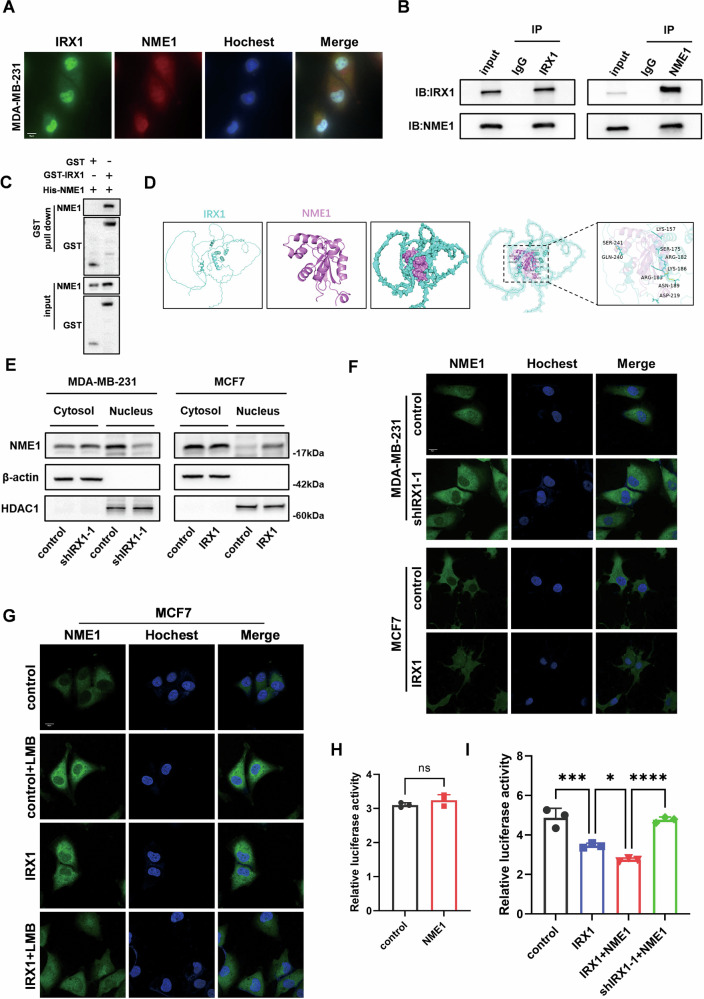
IRX1 recruits NME1 to the nucleus to regulate *ACACA* expression. **A** The intracellular colocalization of IRX1 and NME1 visualized by IF. Scale bar, 16 μm. **B** The interaction between IRX1 and NME1 was verified by co-IP assay. **C** GST-pulldown assay demonstrating a direct interaction between purified IRX1 and NME1 proteins. **D** 3D structure model and interaction of IRX1 and NME1. **E** The NME1 expression level in the nucleus and cytoplasm was detected by nucleocytoplasmic separation assay. **F** The localization of NME1 in *IRX1*-knockdown MDA-MB-231 or *IRX1*-overexpressing MCF7 cells was determined by IF. Scale bar, 16 μm. **G** NME1 localization was observed by IF after treatment of control and IRX1-overexpressing MCF7 cells with Leptomycin B (LMB, 10 ng/μL). Scale bar, 16 μm. **H**, **I** The effect of NME1 or IRX1 on *ACACA* promoter activity was determined by dual-luciferase analysis. *P < 0.05, **P < 0.01, ***P < 0.001 or ****P < 0.0001.

To further elucidate the mechanism of IRX1 function, we performed co-immunoprecipitation coupled with mass spectrometry (coIP-MS). Among the high-confidence interacting proteins identified, NME1 emerged as a top candidate (Fig. S4A). Previous studies indicate that NME1, which shuttles between nuclear and cytoplasmic compartments, can interact with transcription factors and modulate their activity, potentially functioning as a transcriptional co-regulator [27]. We therefore hypothesized that NME1 interacts with IRX1 to facilitate the transcriptional repression of *ACACA*. We first confirmed the IRX1-NME1 interaction using immunofluorescence and co-IP assays. Immunofluorescence revealed significant nuclear co-localization of IRX1 and NME1 (Figs. 6A and S4B), while co-IP demonstrated an interaction between the two proteins in breast cancer cells (Fig. 6B). Furthermore, GST-pull down assay showed that NME1 binds directly to IRX1 (Fig. 6C). Computational analysis and structural modeling using PDBePISA and PyMOL software predicted a potential binding interface between IRX1 and NME1 (Fig. 6D). These findings confirm the interaction between IRX1 and NME1. Notably, IRX1 did not affect the NME1 expression (Fig. S4C, D).

Given NME1’s known nucleocytoplasmic shuttling capability, we investigated whether IRX1 influences its subcellular distribution. Nuclear-cytoplasmic fractionation assays showed that *IRX1* overexpression enriched NME1 in the nuclear fraction, whereas *IRX1* knockdown reduced nuclear NME1 levels (Fig. 6E). Immunofluorescence analysis consistently demonstrated that IRX1 promotes the nuclear accumulation of NME1 (Fig. 6F). Furthermore, a nucleocytoplasmic shuttling blockade assay combined with immunofluorescence analysis revealed that following treatment with Leptomycin B (LMB, 10 ng/μL), the nuclear fluorescence intensity of NME1 in the IRX1 + LMB group was significantly stronger than that in the control + LMB group, indicating that IRX1 primarily alters the nuclear localization of NME1 by promoting its nuclear import rather than inhibiting its nuclear export (Fig. 6G). We next assessed the functional consequence of the IRX1-NME1 interaction on *ACACA* transcription using dual luciferase reporter gene assay. Overexpression of NME1 alone had no effect on *ACACA* promoter activity (Fig. 6H). However, when co-expressed with IRX1, NME1 synergistically enhanced IRX1-mediated repression of the *ACACA* promoter. Conversely, NME1 overexpression failed to influence *ACACA* promoter activity in IRX1-knockdown cells (Fig. 6I). Collectively, these results demonstrate that NME1 directly interacts with IRX1, is recruited by IRX1 to the nucleus, and functions as a co-repressor to enhance IRX1-mediated transcriptional repression of *ACACA*.

### The *IRX1-NME1/ACACA* axis regulates de novo fatty acid synthesis and breast cancer progression

**Fig. 7:**
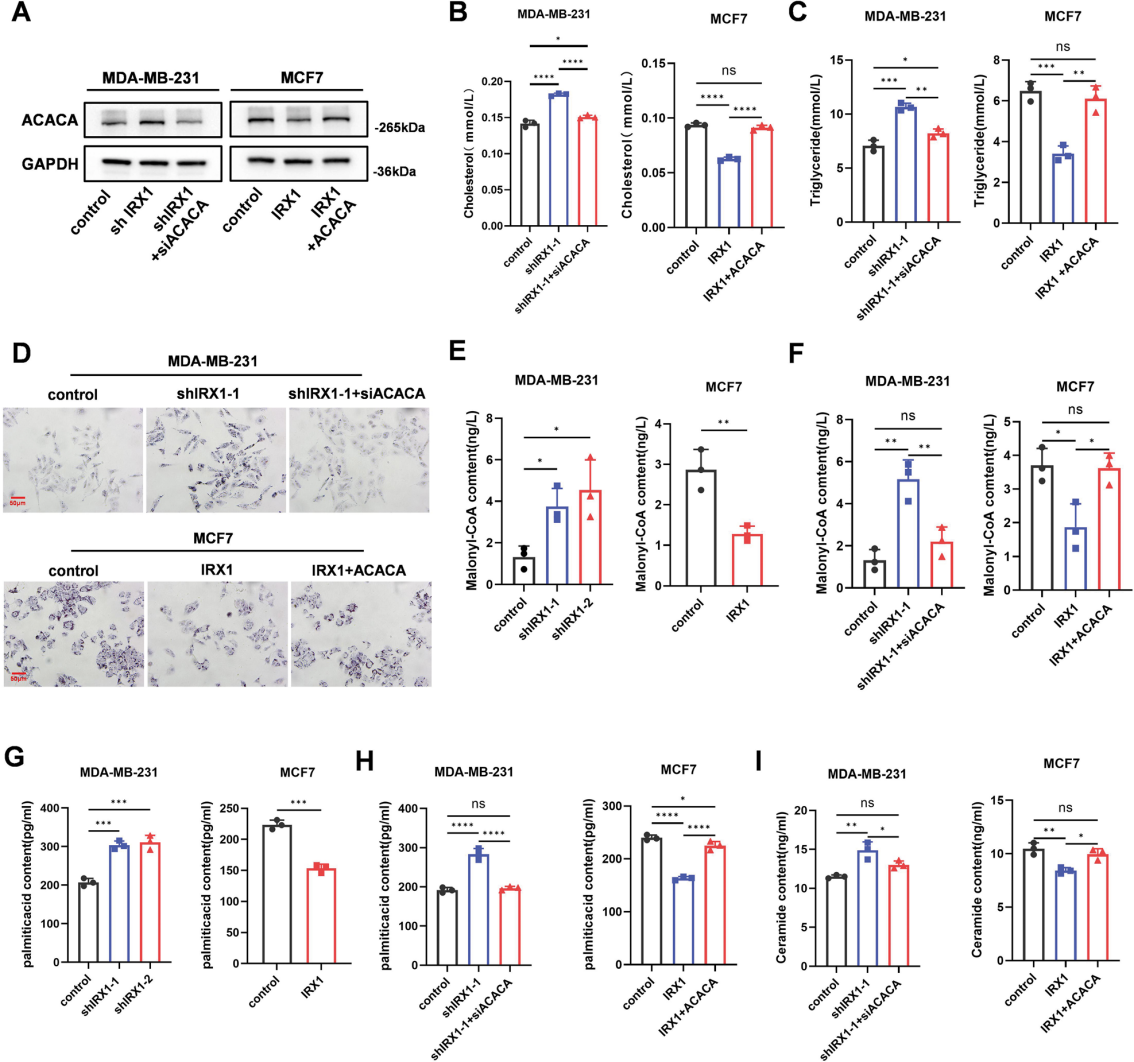
*IRX1-NME1/ACACA* axis regulates de novo fatty acid synthesis in breast cancer. **A** The expression of ACACA in *IRX1/ACACA*-knockdown MDA-MB-231 or *IRX1/ACACA*-overexpressing MCF7 cell was determined by western blot. Measurement of cholesterol (**B**) and triglyceride (**C**) content and oil red O staining (**D**) in cells as in **A**. Scale bar, 50 μm (**D**). The intracellular Malonyl-CoA (**E**, **F**) or palmitate (**G**, **H**) level of cells as in **A** was determined by ELISA. **I** The intracellular ceramide level of cells as in **A** was determined by ELISA. *P < 0.05, **P < 0.01, ***P < 0.001 or ****P < 0.0001.

To determine whether the IRX1-NME1 complex regulates lipid metabolism through *ACACA* in breast cancer, we concurrently knocked down or overexpressed *ACACA* in *IRX1*-knockdown MDA-MB-231 and T47D cells, as well as in *IRX1*-overexpressing MCF7 and CAL51 cells (Figs. 7A and S5A). Measurements of cellular triglyceride and cholesterol levels, along with Oil Red O staining, demonstrated that modulating *ACACA* expression rescued the lipid metabolic changes induced by *IRX1* (Figs. 7B–D and S5B–D). *ACACA* catalyzes the carboxylation of acetyl-CoA to malonyl-CoA, which is the crucial step in palmitate synthesis. We therefore assessed the impact of the *IRX1-NME1/ACACA* axis on these specific metabolites. ELISA measurements showed that *IRX1* overexpression reduced cellular malonyl-CoA and palmitate levels. This reduction was reversed by concurrent *ACACA* overexpression. Conversely, in *IRX1*‑knockdown cells, which exhibited elevated levels of these metabolites, *ACACA* knockdown an abrogated the increase (Figs. 7E–H and S5E, F). To further elucidate the regulatory role of the *IRX1/NME1–ACACA* axis in lipid metabolism in breast cancer cells, we analyzed the effect of *IRX1* on ceramide, a palmitate-derived signaling lipid. Ceramide levels in each group of cells were measured using a ceramide ELISA kit. The results revealed that *IRX1* overexpression reduced intracellular ceramide levels, whereas *IRX1* knockdown led to increased ceramide levels. Similarly, *ACACA* reversed the effect of *IRX1* on ceramide levels (Figs. 7I and S5G). These findings suggest that the IRX1-NME1 complex regulates de novo fatty acid synthesis via ACACA, thereby modulating lipid metabolism in breast cancer cells.

**Fig. 8:**
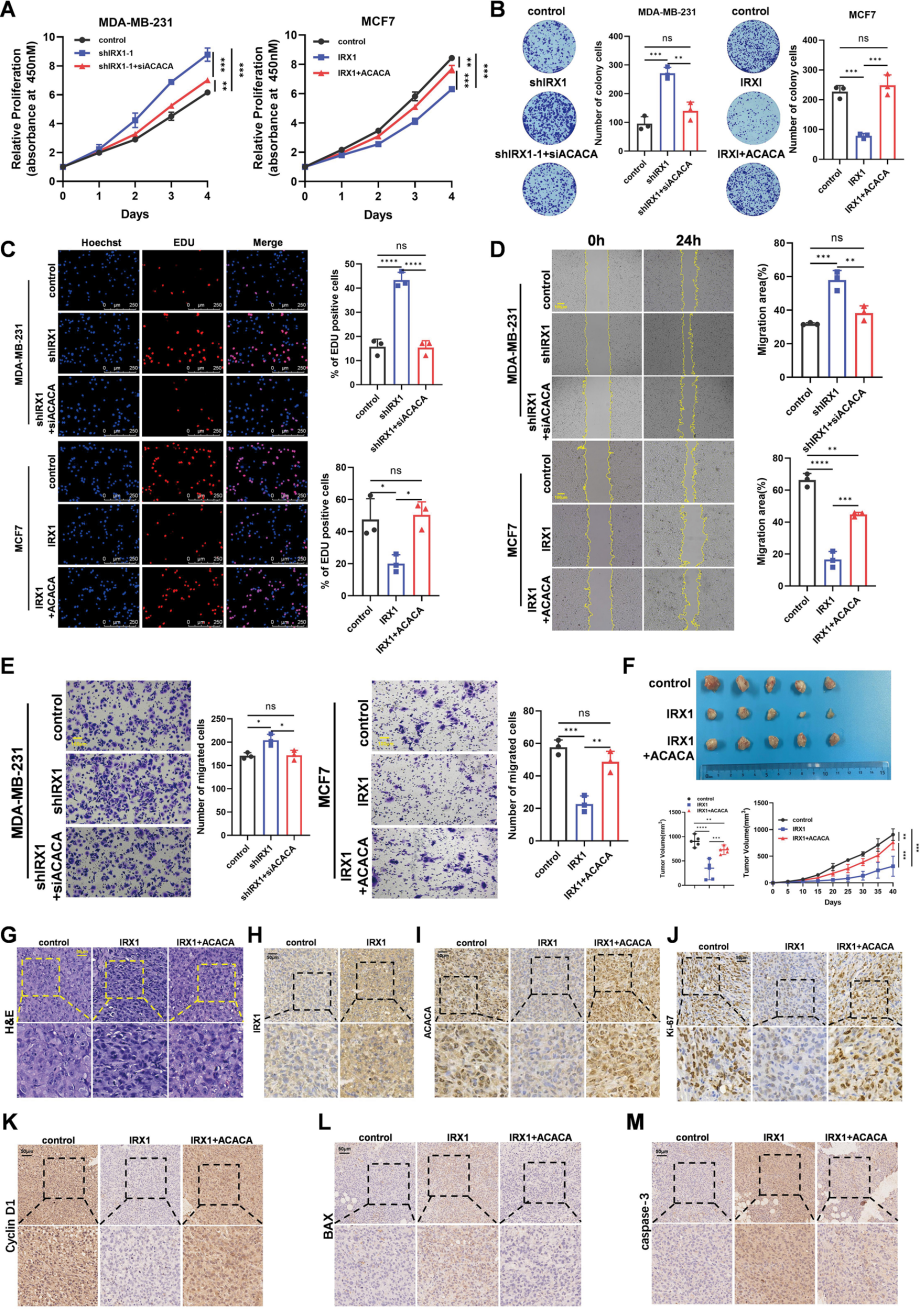
*IRX1-NME1/ACACA* axis affects breast cancer progression in vitro and in vivo*.* CCK8 (**A**), colony formation (**B**), and EdU assays (**C**) were performed to assess the effect of *IRX1/ACACA* on breast cancer proliferation. Scale bar, 250 μm (**C**). Wound healing (**D**) and Transwell (**E**) assays were performed to assess the effect of *IRX1/ACACA* on breast cancer migration. Scale bar, 100 μm (**D**, **E**). **F** Representative images, tumor volume and growth curves of subcutaneous xenografts in NSG mice derived from *IRX1*-overexpressing, *IRX1/ACACA*-overexpressing and control cells (*n* = 5). HE staining (**G**) of mouse tumor tissue sections and the expression levels of IRX1 (**H**), ACACA (**I**), Ki-67 (**J**), Cyclin D1 (**K**), BAX (**L**) and Caspase-3 (**M**) was assessed by IHC staining. Scale bar, 50 μm (**G**, **M**).

CCK8, colony formation, and EDU assays consistently showed that *ACACA* expression rescued the proliferative phenotypes induced by *IRX1* modulation (Figs. 8A–C and S6A–C). Similarly, wound healing and Transwell assays demonstrated that *ACACA* rescued the migratory capacities altered by *IRX1* (Figs. 8D, E and S6D, E). In summary, these rescue experiments demonstrate that the *IRX1-NME1* axis regulates breast cancer proliferation, migration, and de novo fatty acid synthesis by transcriptionally repressing *ACACA*. We further validated the role of *IRX1* and *ACACA* using a xenograft mouse model. NSG mice were subcutaneously injected with control MCF7 cells, *IRX1-*overexpressing MCF7 cells, or *IRX1*-overexpressing MCF7 cells with *ACACA* overexpression (*n* = 5). Tumor volume was significantly reduced in the *IRX1*-overexpressing group compared to controls. In contrast, concurrent *ACACA* overexpression rescued tumor growth, indicating that *ACACA* attenuates the tumor-suppressive effect of *IRX1* in vivo (Fig. 8F). Histological analysis of tumor sections via H&E staining revealed that ACACA overexpression reversed the suppression of tumor cell proliferation caused by IRX1 (Fig. 8G). IHC confirmed reduced ACACA expression in IRX1-overexpressing tumors, and analysis of proliferation and apoptosis-related markers showed that ACACA overexpression reversed the effects of IRX1 on these markers (Fig. 8H–M).

### Upregulation of *ACACA* correlates with poor prognosis in breast cancer

**Fig. 9:**
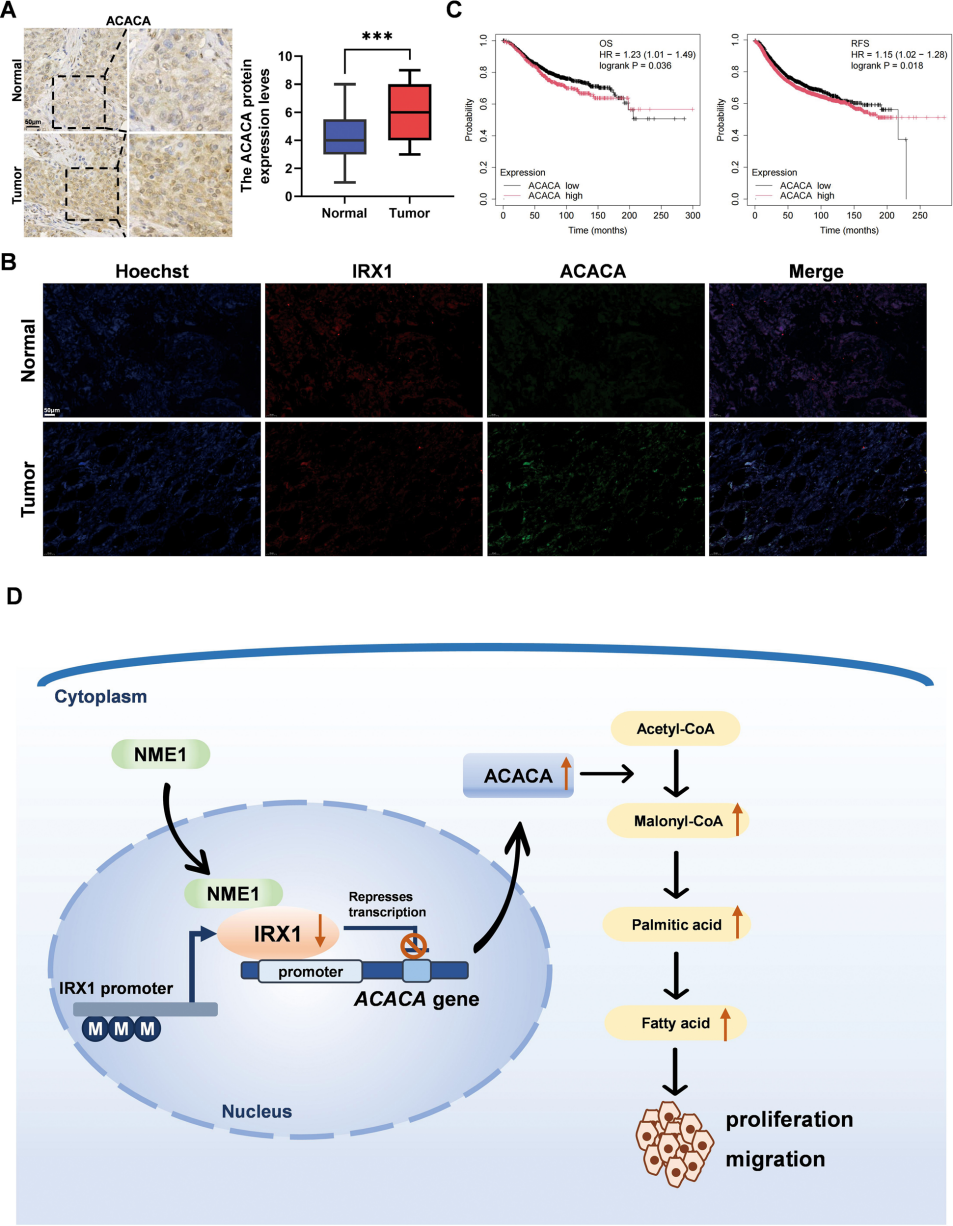
*ACACA* was correlated with the prognosis of breast cancer patients. **A** The protein expression levels of ACACA in breast cancer and normal tissues by IHC staining. Scale bar, 50 μm. **B** The expression of IRX1 and ACACA in breast cancer and normal tissues was detected by immunofluorescence. Scale bar, 50 μm. **C** Kaplan–Meier plotter analysis of OS and RFS in breast cancer patients with different *ACACA* expression levels from the TCGA database. **D** A model of *IRX1-NME1/ACACA* in breast cancer progression. *P < 0.05, **P < 0.01, ***P < 0.001 or ****P < 0.0001.

To further support the role of IRX1 in influencing the malignant progression and prognosis of breast cancer patients through *ACACA*, we performed IHC staining on 20 pairs of clinical specimens. The results confirmed that ACACA protein levels were significantly higher in breast cancer tissues compared to adjacent normal tissues (Fig. 9A). Multiplex immunofluorescence staining of clinical specimens demonstrated a negative correlation between IRX1 and ACACA protein levels in breast cancer tissues (Fig. 9B). Analysis of the KM-plotter database revealed that high *ACACA* expression is significantly associated with poorer OS and PFS in breast cancer patients (Fig. 9C), underscoring its clinical relevance as a prognostic marker. In summary, our findings establish that the *IRX1-NME1/ACACA* axis critically regulates breast cancer progression and de novo fatty acid synthesis (Fig. 9D). This novel identified regulatory axis may represent a promising therapeutic target for breast cancer intervention.

## Discussion

Breast cancer remains a major threat to women worldwide. This study identifies a novel *IRX1-NME1/ACACA* regulatory axis that plays a critical role in de novo fatty acid synthesis and breast cancer progression. We found that the transcription factor IRX1 is significantly downregulated in breast cancer, where it functions as a tumor suppressor by inhibiting malignant progression and is associated with favorable patient prognosis. Mechanistically, *IRX1* transcriptionally represses *ACACA* —a rate-limiting enzyme in de novo fatty acid synthesis—by directly binding to its promoter, thereby attenuating de novo fatty acid synthesis in breast cancer cells. Furthermore, we demonstrate that IRX1 physically interacts with the tumor suppressor NME1 and facilitates its nuclear translocation. Nuclear NME1 subsequently acts as a co-repressor to enhance *IRX1*-mediated transcriptional repression of *ACACA*. Collectively, our findings elucidate the mechanism of the *IRX1-NME1/ACACA* axis in breast cancer and highlight its potential as a therapeutic target for novel treatment strategies.

Reprogrammed lipid metabolism is a hallmark of cancer, as cancer cells exploit this process to generate energy, membrane components, and signaling molecules that fuel their proliferation, invasion, and metastasis [28]. Fatty acids are central to lipid metabolism, and their de novo synthesis is governed by Acetyl-CoA Carboxylase Alpha (ACACA), the rate-limiting enzyme. ACACA catalyzes the carboxylation of acetyl-CoA to malonyl-CoA. Fatty acid synthase (FASN) then utilizes malonyl-CoA to produce palmitate, the precursor for more complex lipids including triglycerides and phospholipids [29, 30]. *ACACA* is overexpressed in numerous cancers (e.g., lung, colorectal, breast, prostate, liver). It promotes hepatocellular carcinoma proliferation by activating CCNA2, CCND1, MYCN, and JUN, whereas knockdown of ACACA impairs fatty acid production and induces apoptosis in prostate cancer [31, 32]. In ER+ breast cancer, ACACA confers resistance to anti-estrogen therapy, suggesting its targeting could restore treatment sensitivity [33]. *ACACA* is regulated at multiple levels. Transcriptionally, circCAPRIN1 activates *ACACA* via STAT2 in colorectal cancer, while SREBP1 mediates its upregulation upon PTPRO silencing [21, 34]. Post-transcriptionally, CDK13-driven m5C modification stabilizes *ACACA* mRNA in prostate cancer [24]. Post-translationally, AMPK phosphorylates ACACA at Ser79 to inhibit its enzymatic activity [35]. Our study unveils *IRX1* as a novel transcriptional repressor of *ACACA*, thereby curtailing de novo fatty acid synthesis and impeding breast cancer progression. The upstream signaling pathways governing *IRX1* itself warrant future investigation.

The metastasis suppressor NME1 is downregulated in multiple cancer types, including breast, melanoma, liver, and colon cancers, in which its low expression correlates with increased metastatic potential [36–41]. Extracellular vesicles carrying NME1, released by breast cancer cells, can modulate genes involved in fatty acid and cholesterol metabolism in fibroblasts, implicating NME1 in intercellular metabolic regulation within the tumor microenvironment [42]. Initially, NME1 was considered to be primarily a cytoplasmic protein; however, immunohistochemistry and subcellular fractionation later confirmed its presence in the nucleus. Nevertheless, NME1 lacks a classical nuclear localization signal, and the mechanisms underlying its nuclear translocation remain poorly understood. A review article has suggested that, due to the high proportion of negatively charged amino acid residues on its conserved membrane-binding domain, NME1 is unable to associate with lipid membranes. Additionally, its low surface hydrophobicity suggests that nuclear transport of NME1 requires active transport mediated by physical interactions with other proteins and lipids of the nuclear membrane [27]. At present, only one study has demonstrated that the interaction between NME1 and the viral antigen EBNA3C promotes the nuclear localization of NME1 [43]. In this study, we found that IRX1 interacted with NME1 and affected its nuclear localization.

NME1 has been shown to play a role in transcriptional regulation [44]. NME1 binds to the NHEs region within the PDGFA promoter, thereby repressing PDGFA expression. Additionally, studies have revealed that the promoter region of TP53 also contains potential binding sites for NME1, which competes with IFI16 for binding to the G-rich region of the TP53 promoter, thereby influencing its transcriptional activity [45]. Furthermore, NME1 can physically interact with other transcription factors and may function as a transcriptional coactivator or corepressor, thereby regulating the expression of genes involved in tumor proliferation and migration [27, 43, 46–49]. As demonstrated by Choudhuri et al., NME1 indirectly suppresses cyclin D1 expression through physical interaction with the transcription factor AP1, leading to B-cell cycle arrest and TP53-dependent apoptosis [46]. We discovered that IRX1 interacts with NME1 without altering its total protein levels. Instead, IRX1 facilitates the nuclear translocation of NME1, which then functions as a co-repressor to augment IRX1-mediated transcriptional repression of *ACACA*.

Epigenetic mechanisms, particularly DNA methylation, are crucial in breast cancer. For example, *EZH2* methylation is linked to metastasis [50], and *DRD2* promoter methylation modulates NF-κB signaling [51]. *IRX1* expression is likewise subject to epigenetic silencing. In gastric cancer, PRMT5 recruits DNMT3A to hypermethylate the *IRX1* promoter [11], while in head and neck squamous cell carcinoma, *IRX1* hypermethylation downregulates its expression and perturbs TGF-β signaling [12, 13]. Our bioinformatic analysis predicted a CpG island in the *IRX1* promoter. We subsequently confirmed *IRX1* promoter hypermethylation in clinical breast cancer specimens and cell lines by bisulfite sequencing PCR (BSP), which inversely correlated with *IRX1* transcript levels.

Dysregulation of transcription factors is a well-established driver of tumor progression. Transcription factors can function as either oncogenes or tumor suppressors, orchestrating diverse cellular processes—including proliferation, differentiation, apoptosis, metabolism, invasion, and metastasis—through the regulation of target gene expression. For instance, *RUNX2* recruits the NuRD/CRL4B complex to promote breast cancer bone metastasis [52], whereas *CBFB* cooperates with *RUNX1* to inhibit the *NOTCH3* pathway, suppressing tumorigenesis [53]. Similarly, *OVOL2* acts as a transcriptional repressor of aerobic glycolysis, attenuating the Warburg effect and tumor malignancy [54]. Emerging therapeutic strategies, such as proteolysis-targeting chimeras (PROTACs) and cysteine-reactive inhibitors, are being developed to target transcription factors, highlighting their potential as promising targets for precision oncology [55]. *IRX1* encodes a homeodomain-containing transcription factor implicated in embryonic development and tissue differentiation. *IRX1* acts as a tumor suppressor in gastric, lung, and esophageal cancers, where it inhibits proliferation and migration while promoting apoptosis of cancer cells [9–11, 25, 56]. Previous studies on *IRX1* in breast cancer have been limited. Only one report has shown that downregulation of *TET1* impairs demethylation of the *IRX1* promoter, leading to *IRX1* silencing and contributing to tumor progression in invasive ductal carcinoma [26]. However, the specific role of *IRX1* in breast cancer was not elucidated in that study. In contrast, our study identifies a tumor-suppressive role for *IRX1* in breast cancer, demonstrating its impact on malignant progression both in vitro and in vivo.

The molecular mechanisms by which *IRX1* exerts its functions in various tumor types are emerging. In gastric cancer, *IRX1* suppresses peritoneal dissemination and distant metastasis by inhibiting BDKRB2-dependent angiogenesis and vasculogenic mimicry [10]. In osteosarcoma, *IRX1* promotes cell invasion and metastasis by upregulating CXCL14/NF-κB signaling pathway [14]. In head and neck squamous cell carcinoma, *IRX1* is epigenetically silenced and interferes with the TGF-β signaling pathway, leading to decreased apoptosis and increased proliferation [12, 13]. Previous studies have largely focused on the effects of *IRX1* on tumor-related signaling pathways, specifically regarding the expression of relevant proteins and the consequent impact on malignant progression. Differing from these approaches, our study employed transcriptomic and metabolomic sequencing to reveal that *IRX1* participates in lipid metabolism in breast cancer cells by regulating the expression of lipid metabolism-related genes. Furthermore, a series of lipid metabolism-related experiments confirmed the inhibitory effect of *IRX1* on lipid accumulation in breast cancer cells. Breast cancer originates within a lipid-rich microenvironment and is highly dependent on lipid metabolic reprogramming to fulfill its growth and progression requirements [57]. Our findings refine the regulatory framework of fatty acid metabolism in breast cancer and provide a novel theoretical basis for developing therapeutic strategies targeting lipid metabolism in this malignancy. Furthermore, our study demonstrates for the first time that *IRX1* directly regulates the transcriptional activity of *ACACA*, revealing the critical role of *IRX1* in the fatty acid metabolism pathway. This discovery not only expands the understanding of *IRX1*’s biological functions but also, more importantly, directly links *IRX* family members to the transcriptional regulation of lipid metabolism for the first time, providing strong evidence for the involvement of the *IRX* gene family in the regulation of lipid metabolism and providing a new perspective for understanding the complex regulatory network of lipid metabolism.

## Supplementary information

supplementary material files

Original data

## Data Availability

All data supporting the findings of this study are available from the corresponding authors upon reasonable request.
