# Supplementary material for: IRX1 suppresses breast cancer progression by inhibiting fatty acid de novo synthesis through downregulating ACACA expression
Source: Cell Death Dis. 2026 Jun 1;17(1):676. doi: 10.1038/s41419-026-08913-9 (PMC13434779; doi:10.1038/s41419-026-08913-9)
Supplement: Supplementary file 1 [file 41419_2026_8913_MOESM2_ESM.pdf]

Original data-western blots

Figure 1E

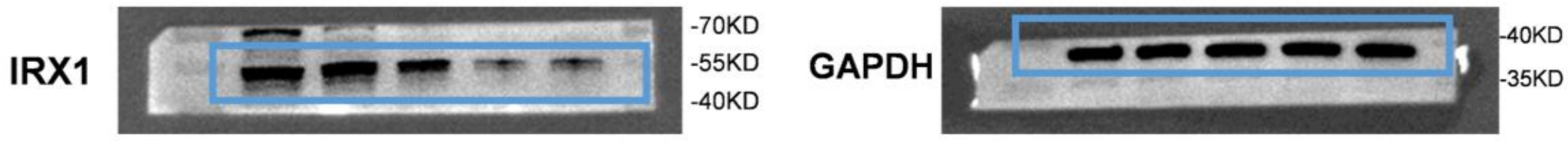

Figure 2E

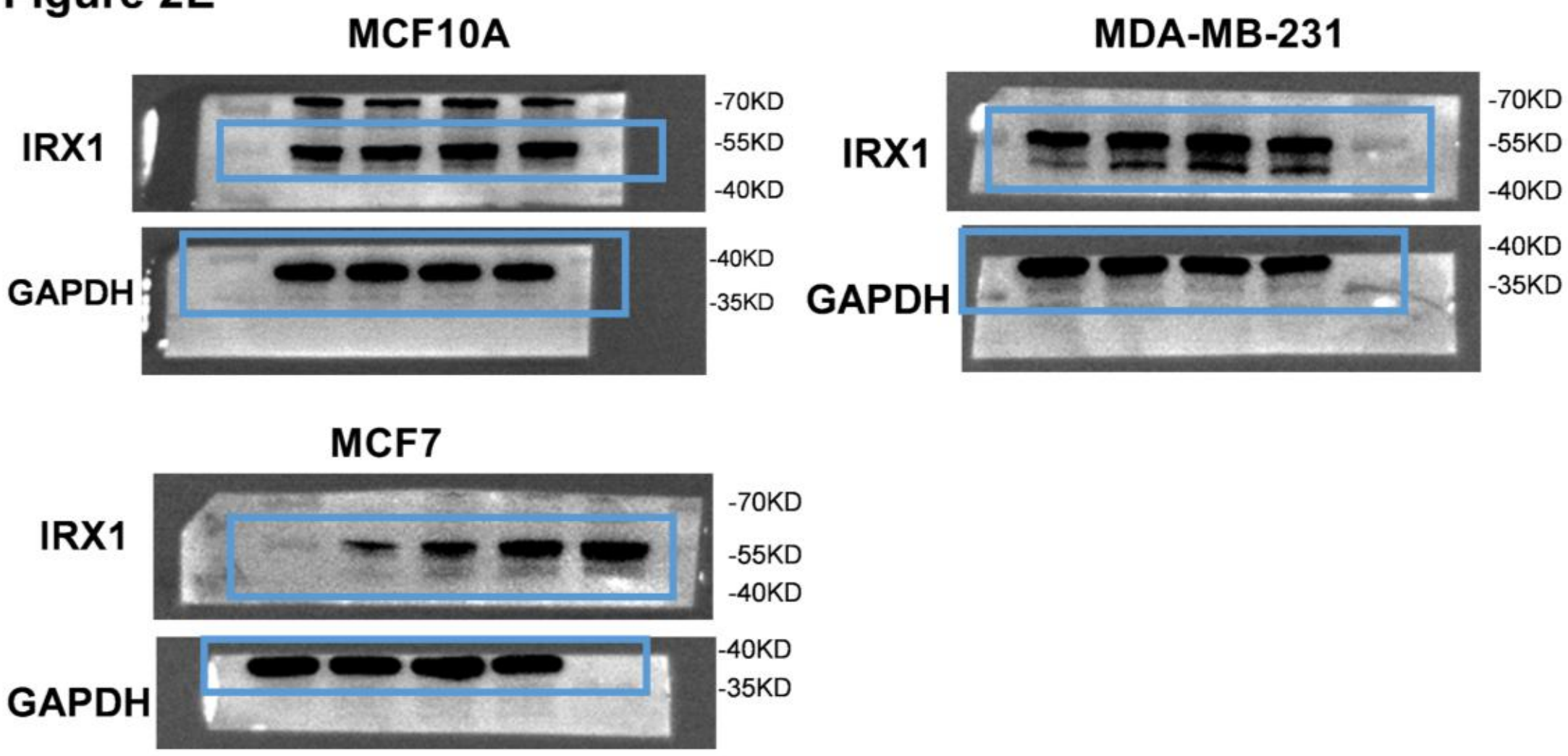

Figure 3A

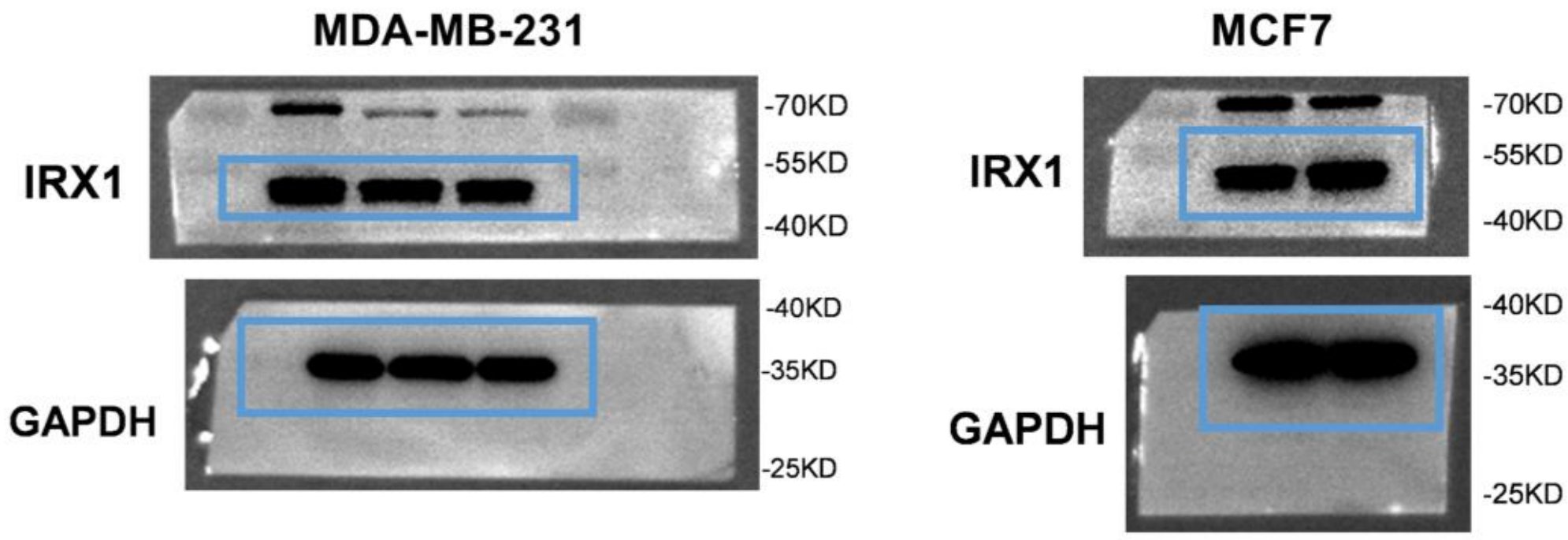

Figure 4E

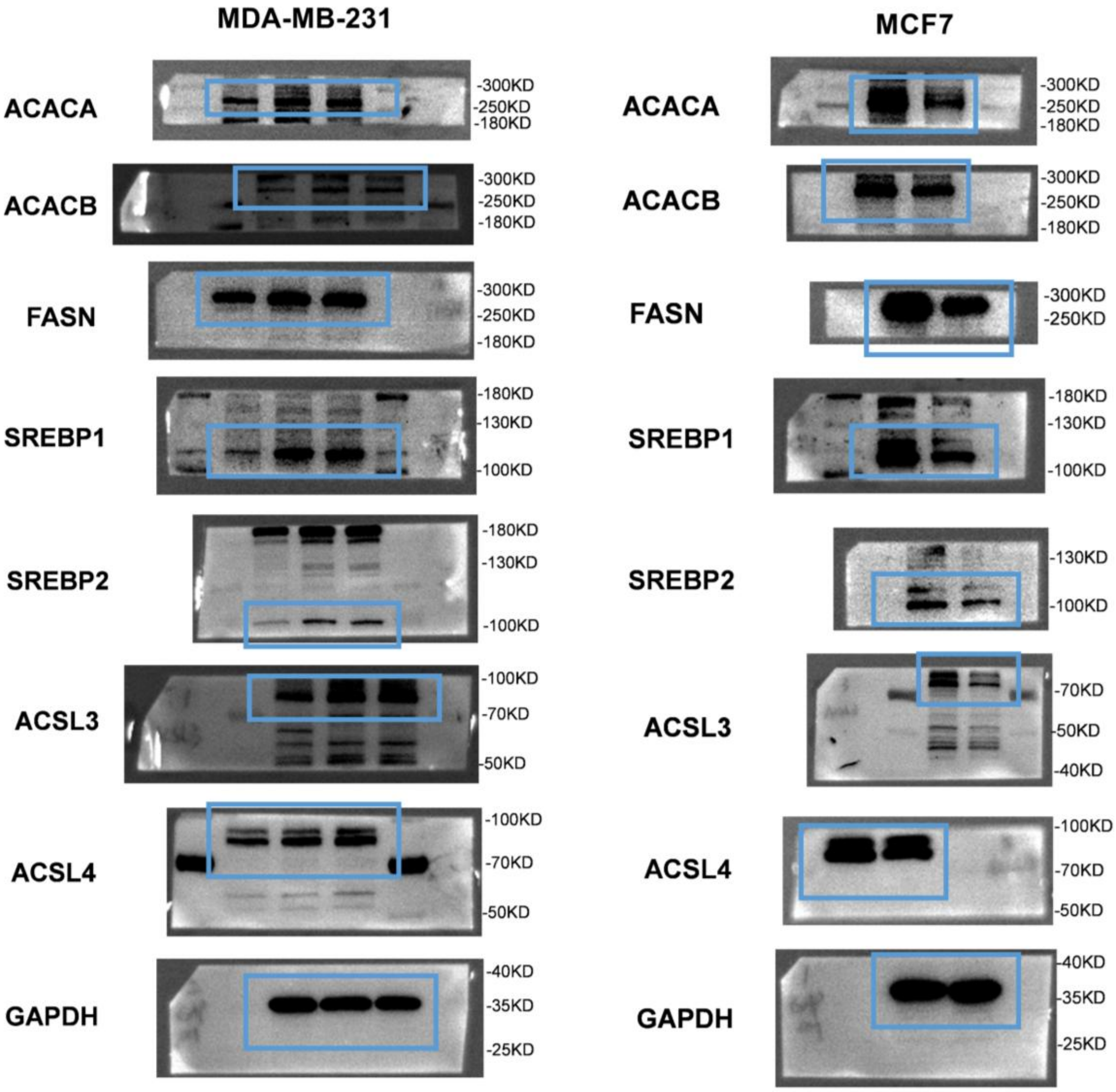
MDA-MB-231
MCF7
ACACA
ACACB
FASN
SREBP1
SREBP2
ACSL3
ACSL4
GAPDH
-300KD
-250KD
-180KD
-130KD
-100KD
-70KD
-50KD
-40KD
-35KD
-25KD

Figure 5H

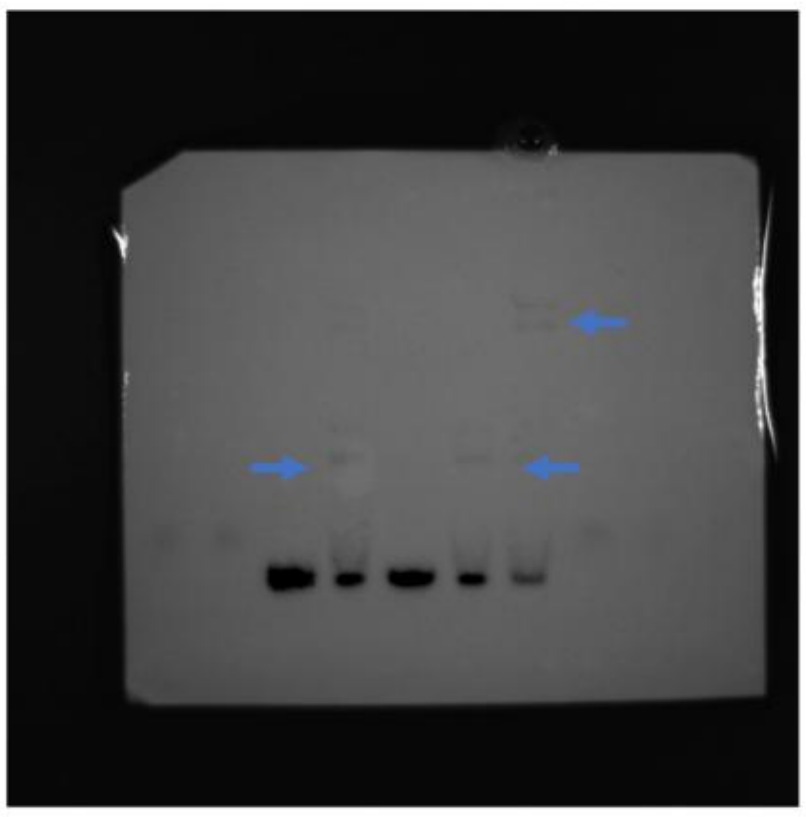

Figure 6B

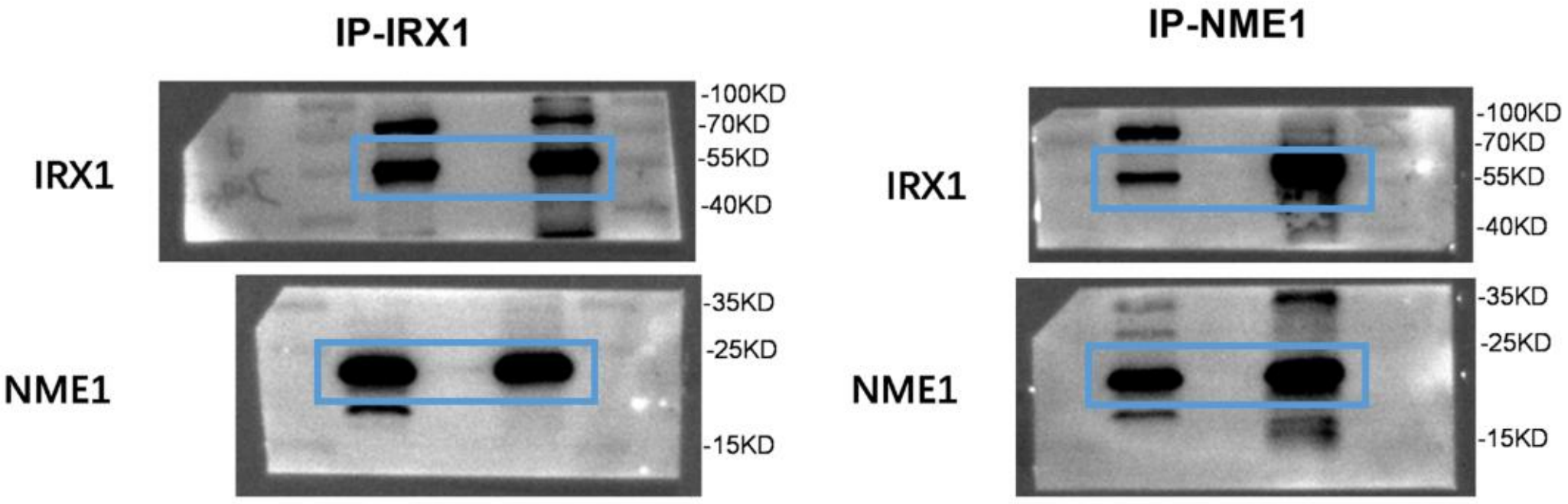

Figure 6C

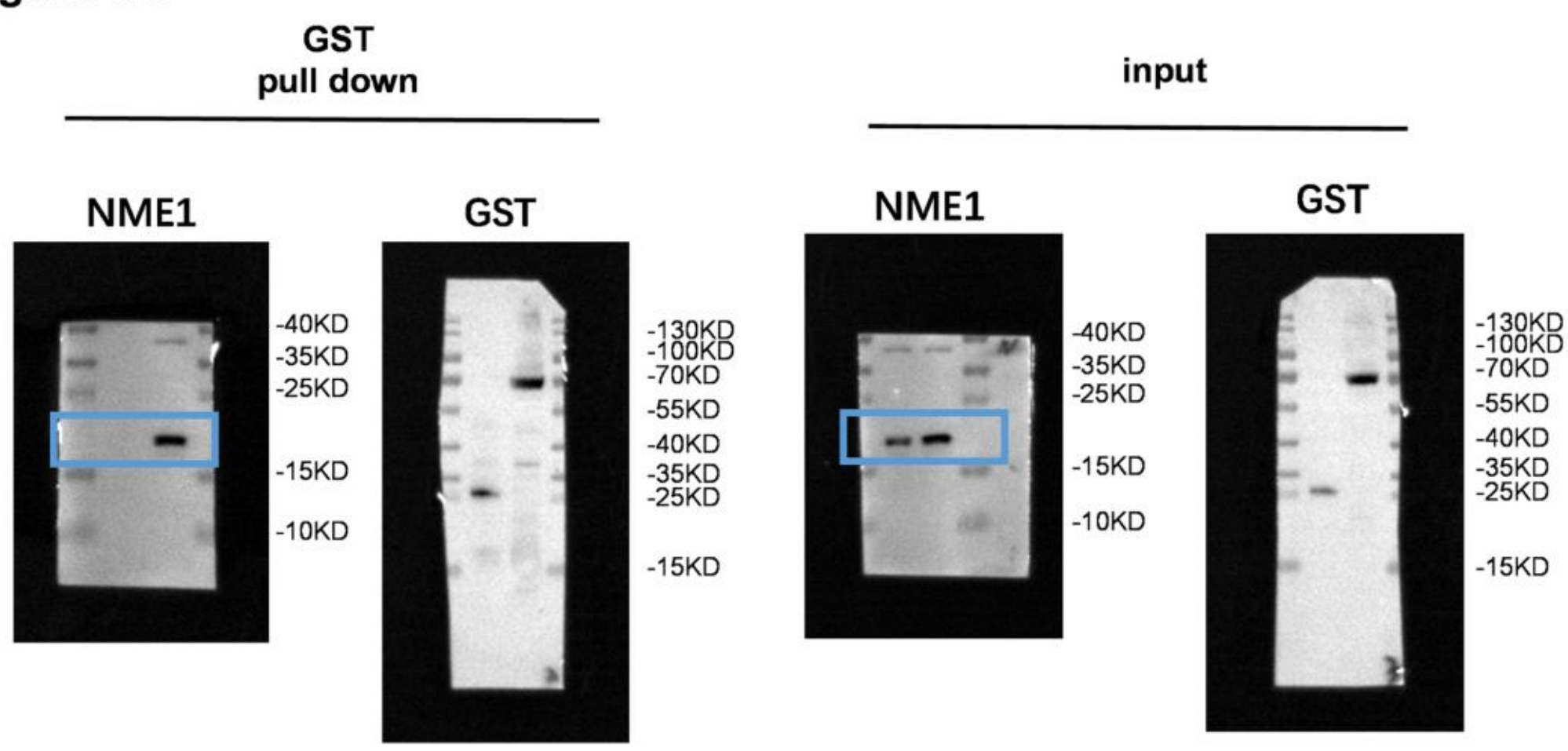

Figure 6E

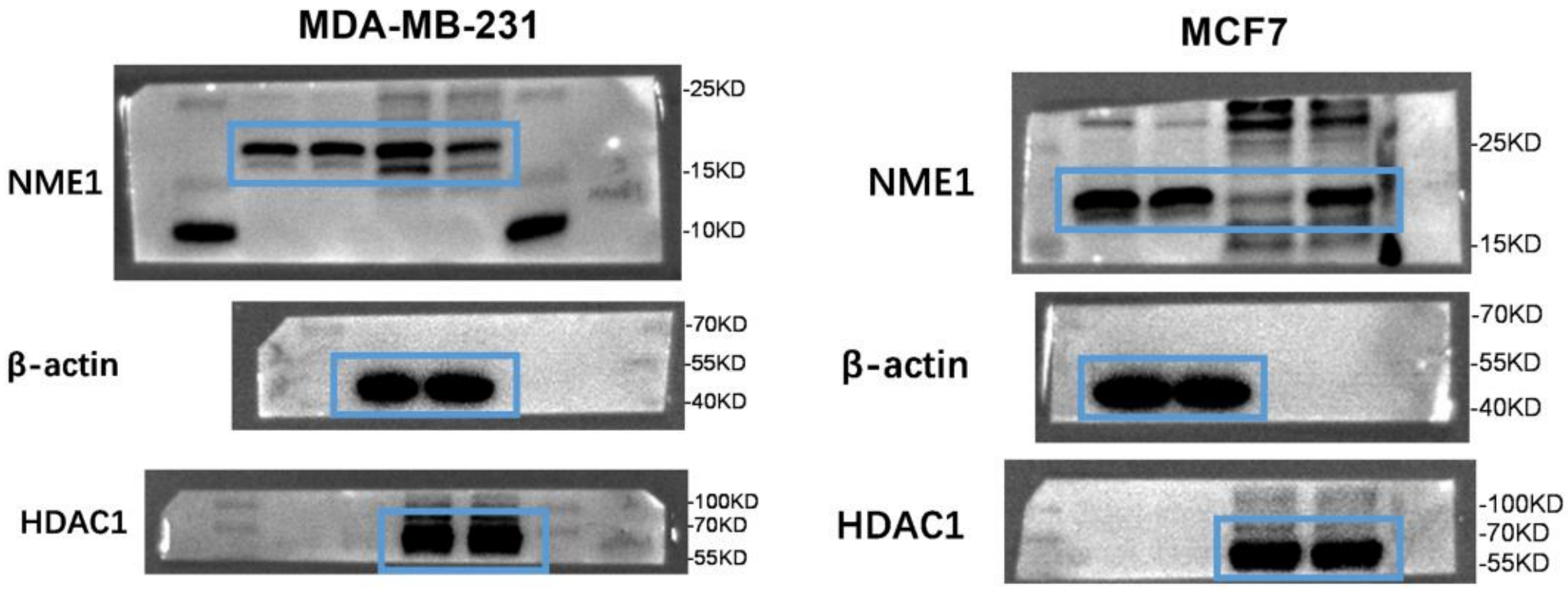

Figure 7A

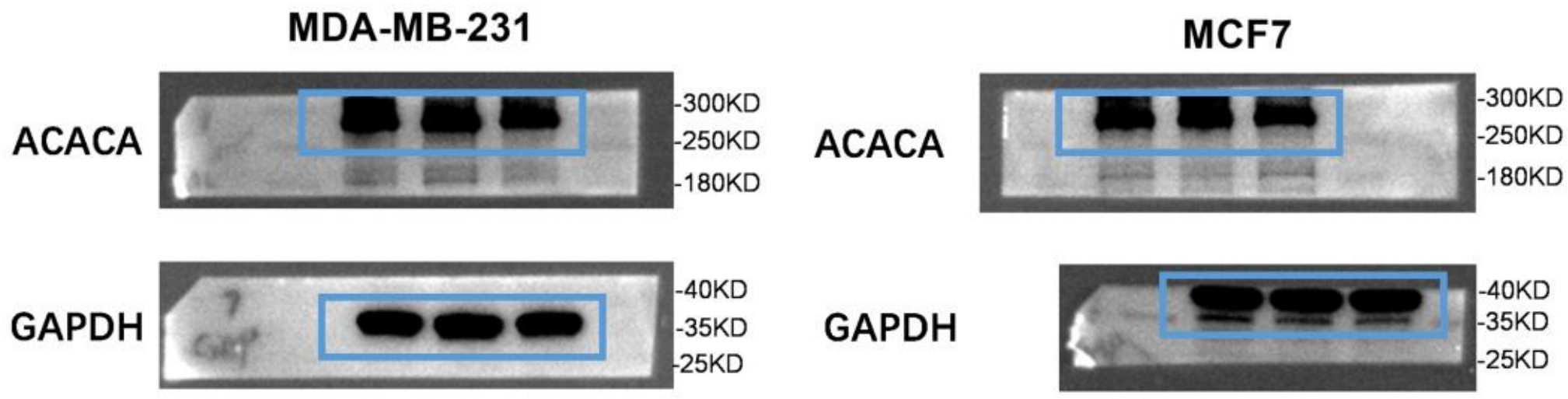

Figure S2C

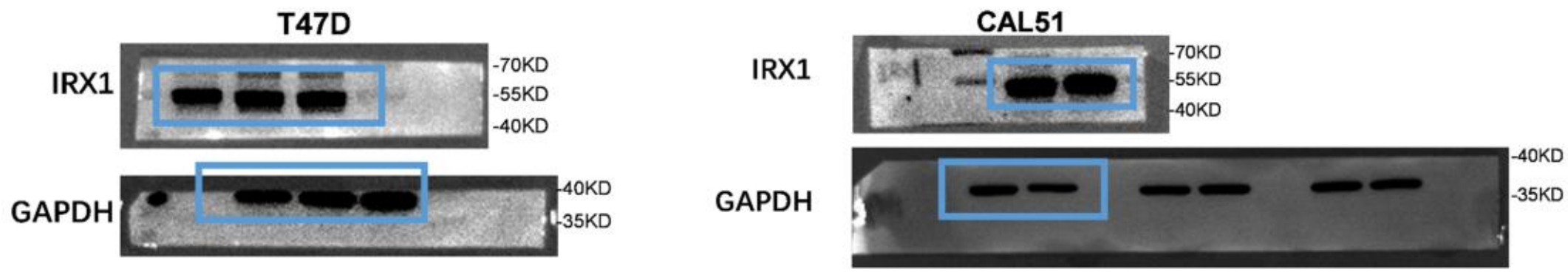

Figure S3C

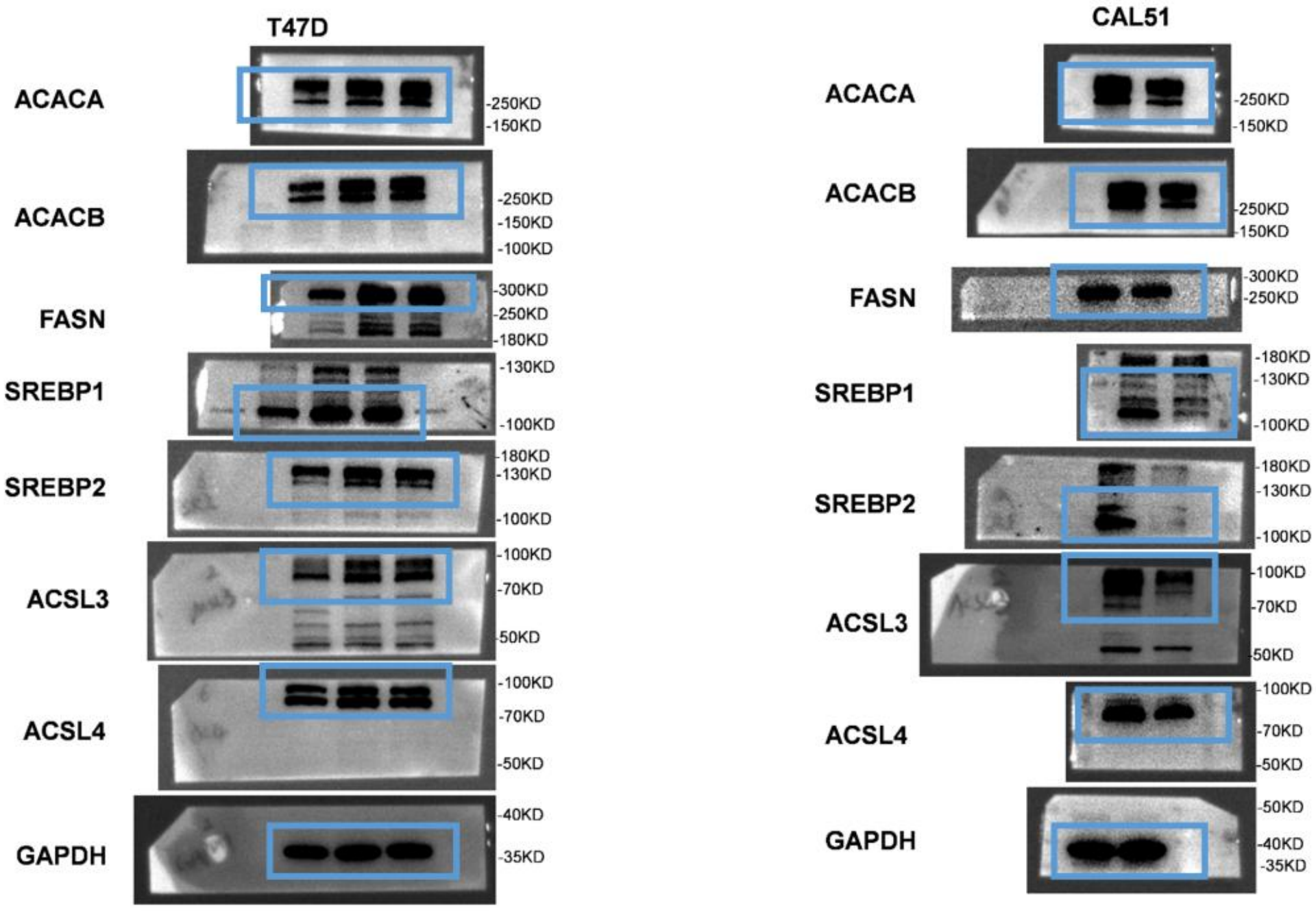

Figure S4D

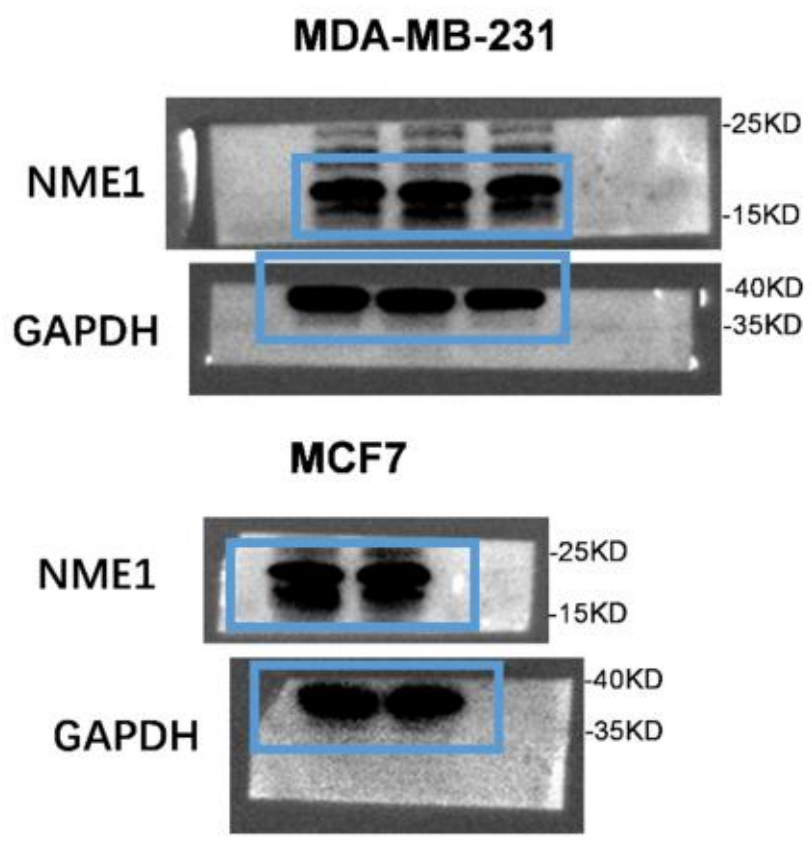

Figure S5A

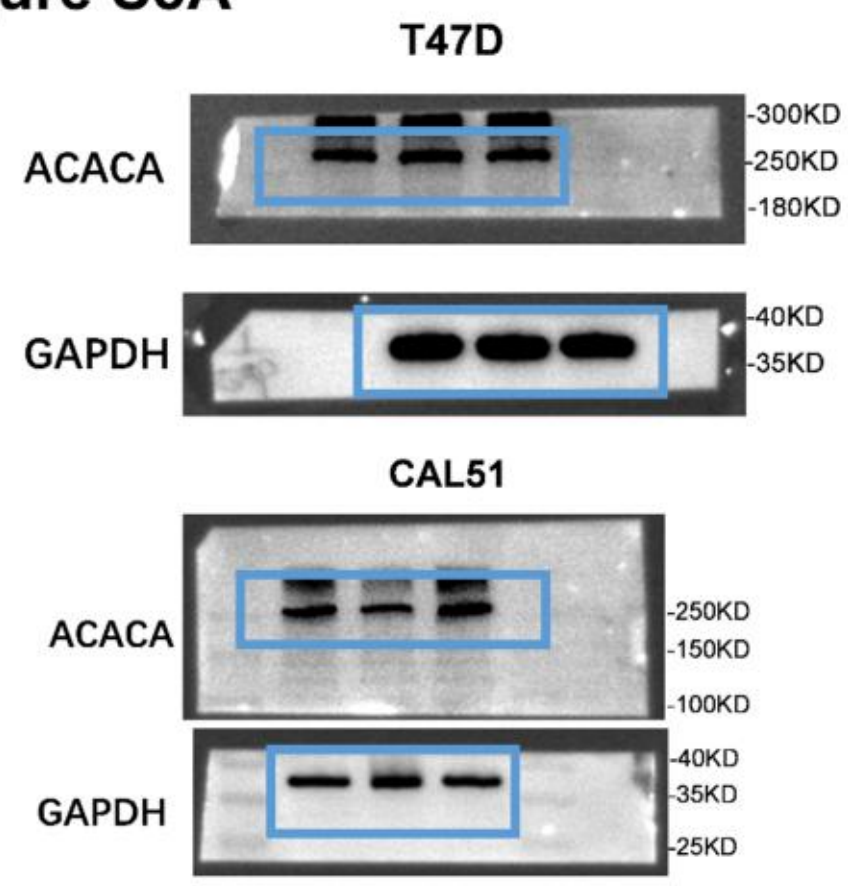

Original data - RT-qPCR

Figure 1H

	MCF10A	MDA-MB-231	T47D	MCF7	CAL51
	1.120296027	0.863638821	0.54554874	0.316604427	0.394683104
IRX1	0.963442185	0.696241638	0.522922844	0.316148293	0.455283369
	0.926491728	0.793278855	0.658607114	0.363945248	0.401467934

Figure 2E

	0(μM)	1(μM)	2(μM)	3(μM)
	1.195543536	1.245737416	1.345989044	1.191682575
IRX1	0.978515539	0.927873476	1.432630604	1.464085696
	0.854804653	1.101905116	1.314638979	1.363202607

Figure 2F

	0(μM)	1(μM)	2(μM)	3(μM)
IRX1	0.937354497	1.480071289	1.785093943	2.225300241
	0.957050307	1.420763739	2.076278541	2.242332156
	1.114708637	1.576436072	2.100889088	2.150988781

Figure 2G

	0(μM)	1(μM)	2(μM)	3(μM)
	0.957271458	1.380955343	1.504899002	1.975201723
IRX1	1.026689546	1.242001285	1.621879534	2.265767771
	1.017479692	1.322865758	1.745531832	2.339175328

Figure 5D

	control	shIRX1-1	shIRX1-2		control	IRX1
	0.893637728	1.486399581	1.391006023		0.859127009	0.125979
ACACA	1.194019171	1.692530697	2.061906148	ACACA	1.031006432	0.154793
	0.937189048	1.825285324	1.546126827		1.128967013	0.271074

Figure S2A

	control	shIRX1-1	shIRX1-2		control	IRX1
	1.028137581	0.066915458	0.088559446		0.989620072	2.429877
IRX1	0.755316565	0.079788981	0.120858429	IRX1	1.163125416	2.245448
	1.287715005	0.064846986	0.082575064		0.868770287	1.867765

Figure S2B

	control	shIRX1-1	shIRX1-2		control	IRX1
	1.052222156	0.241931859	0.228836539		0.98043009	2.133605
IRX1	1.082989558	0.334600769	0.207844912	IRX1	0.864511264	1.820486
	0.877542762	0.196648715	0.197745851		1.179811737	1.98787

Figure S4C

	control	shIRX1-1	shIRX1-2		control	IRX1
	0.941139182	0.957585581	1.344804695		0.881571812	1.537987
NME1	0.983345293	0.842286725	1.048107479	NME1	0.893940057	1.425693
	1.080538143	0.929227228	0.841565033		1.268919018	0.977788
