# Supplementary material for: IRX1 suppresses breast cancer progression by inhibiting fatty acid de novo synthesis through downregulating ACACA expression
Source: Cell Death Dis. 2026 Jun 1;17(1):676. doi: 10.1038/s41419-026-08913-9 (PMC13434779; doi:10.1038/s41419-026-08913-9)
Supplement: Supplementary file 2 [file 41419_2026_8913_MOESM1_ESM.docx]

**Supplemental data**

**Figure S1. The expression pattern of *IRX* family in human cancers.**

**A)** The *IRX*s expression levels in multiple cancer types analyzed by Oncomine.

**B)** The *IRX*s expression levels in breast cancer visualized by GEPIA.

**C)** OS, RFS and DMFS curves of patients with breast cancer under different *IRX*s expression levels

**Figure S2. *IRX1* inhibits proliferation and migration *in vitro* and *in vivo*.**

**A and B)** The mRNA expression level of *IRX1* in *IRX1-*konckdown MDA-MB-231 (A) and T47D (B) cells, or *IRX1*-overexpressing MCF-7 (A) and CAL51 (B) cells, as well as control cells determined by RT-qPCR.

**C)** The expression of IRX1 in *IRX1*-konckdown T47D, or *IRX1*-overexpressing CAL51 cells, as well as control cells determined by western blot.

**D**-**F)** CCK8 (D), colony formation (E), and EdU assays (F) were performed to assess the effect of *IRX1* on breast cancer proliferation in T47D and CAL51 cells. Scale bar, 250 μm (F).

**G**-**H)** Transwell (**G**) and wound healing (**H**) assays were performed to assess the effect of IRX1 on breast cancer migration in T47D and CAL51 cells.  Scale bar, 100 μm (G and H).

**I)** Representative images, tumor volume and growth curves of subcutaneous xenografts in NSG mice derived from IRX1-knockdown and control cells. (n=5). *P < 0.05, ***P* < 0.01, ****P* < 0.001 or *****P* < 0.0001.

**Figure S3. *IRX1* suppresses lipid metabolism in breast cancer cells**

**A)** GO analysis of differentially expressed genes.

**B)** KEGG analysis of differentially expressed genes.

**C)** The effect of *IRX1* on the expression of lipid metabolism-related genes in T47D and CAL51 cells. determined by western blot.

**D**-**F)** Measurement of oil red O staining (D), cholesterol (E) and triglyceride (F) content in *IRX1*-konckdown T47D or *IRX1*-overexpressing CAL51 cells.  Scale bar, 50 μm(D). **P* < 0.05, ***P* < 0.01, ****P* < 0.001 or *****P* < 0.0001.

**Figure S4. IRX1 interacts with NME1**

**A)** The top 10 interacting genes identified by mass spectrometry.

**B)** Intracellular colocalization of IRX1 and NME1 visualized by IF. Scale bar, 16 μm.

**C**-**D)** RT-qPCR(C) and WB(D) verified that IRX1 did not affect the expression level of NME1.

**Figure S5. *IRX1-NME1/ACACA* axis regulates de novo fatty acid synthesis in breast cancer**

**A)** The expression of ACACA in *IRX1/ACACA*-knockdown T47D or *IRX1/ACACA*-overexpressing CAL51 cell was determined by western blot.

**B-D)** Oil red O staining (B) and measurement of cholesterol (C) and triglyceride (D) content in cells as in (A). Scale bar, 50 μm(B).

**E-F)** The intracellular Malonyl-CoA (E) or palmitate (F) level of cells as in (A) was determined by ELISA.

**G)** The intracellular ceramide level of cells as in (A) was determined by ELISA. **P* < 0.05, ***P* < 0.01, ****P* < 0.001 or *****P* < 0.0001.

**Figure S6. *IRX1-NME1/ACACA* axis affects breast cancer proliferation and migration *in vitro***

**A**-**C)** CCK8 (A), colony formation (B), and EdU assays (C) were performed to assess the effect of *IRX1/ACACA* on breast cancer proliferation. Scale bar, 250 μm(C).

**D**-**E)** Transwell (D) and wound healing (E) assays were performed to assess the effect of *IRX1/ACACA* on breast cancer migration. Scale bar, 100 μm(D-E). **P* < 0.05, ***P* < 0.01, ****P* < 0.001 or *****P* < 0.0001.

**Table S1. Oligonucleotides of siRNAs**

| **siACACA-F** | **GGAACAGUUCAGGAGCUAATT** |
| --- | --- |
| **siACACA-R** | **UUAGCUCCUGAACUGUUCCTT** |
| **siNME1-F** | **CUGAGGAACUGGUAGAUUATT** |
| **siNME1-R** | **UAAUCUACCAGUUCCUCAGTT** |

**Table S2. Sequence for shRNA (5’ to 3’).**

| **shIRX1-1-F** | **CCGGTCTCAGCCTCTTCTCGCAGATCTCGAGATCTGCGAGAAGAGGCTGAGATTTTTG** |
| --- | --- |
| **shIRX1-1-R** | **AATTCAAAAATCTCAGCCTCTTCTCGCAGATCTCGAGATCTGCGAGAAGAGGCTGAG** |
| **shIRX1-2-F** | **CCGGCATTGACAAGATCGACGAGCACTCGAGTGCTCGTCGATCTTGTCAATGTTTTTG** |
| **shIRX1-2-R** | **AATTCAAAAACATTGACAAGATCGACGAGCACTCGAGTGCTCGTCGATCTTGTCAATG** |

**Table S3. Sequence for Luciferase reporter mutation plasmid (5’ to 3’).**

| **PGL3-ACACA-mut-F** | **CTGATCAACACTCGAGAGATCTCAGTAGGAGGCCGGGTGC** |
| --- | --- |
| **PGL3-ACACA-mut-R** | **CCCGGCCTCCTACTGAGATCTCTCGAGTGTTGATCAGTCCC** |

**Table S4. Sequence for methylation-specific PCR (5’ to 3’).**

| **BSP -F** | **GCTTTCTGGGTGAAGAGAAAGC** |
| --- | --- |
| **BSP -R** | **CTCCAGGGGACATCTTTGGCAGAA** |

**Table S5. Sequence used for qPCR (5’ to 3’).**

| **IRX1-F** | **GCTCTTCGGCAGCGACAC** |
| --- | --- |
| **IRX1-R** | **GCTCTGGGGCCTCCTTTG** |
| **NME1-F** | **AAGGAGATCGGCTTGTGGTTT** |
| **NME1-R** | **CTGAGCACAGCTCGTGTAATC** |
| **ACACA-F** | **ATGTCTGGCTTGCACCTAGTA** |
| **ACACA-R** | **CCCCAAAGCGAGTAACAAATTCT** |

**Table S6. Sequence used for EMSA (5’ to 3’).**

| **ACACA-EMSA-F** | **GATCAACACTCGAAAAAGCACAGTAGGAGG** |
| --- | --- |
| **ACACA-EMSA-R** | **CCTCCTACTGTGCTTTTTCGTGTGTTGATC** |
| **ACACA-EMSA-mut-F** | **GATCAACACTCGAGAGATCTCAGTAGGAGG** |
| **ACACA-EMSA-mut-R** | **CCTCCTACTGTGCTCTCTAGAGTGTTGATC** |
